# PHD1 regulates p53-mediated colorectal cancer chemoresistance

**DOI:** 10.15252/emmm.201505492

**Published:** 2015-08-19

**Authors:** Sofie Deschoemaeker, Giusy Di Conza, Sergio Lilla, Rosa Martín-Pérez, Daniela Mennerich, Lise Boon, Stefanie Hendrikx, Oliver DK Maddocks, Christian Marx, Praveen Radhakrishnan, Hans Prenen, Martin Schneider, Johanna Myllyharju, Thomas Kietzmann, Karen H Vousden, Sara Zanivan, Massimiliano Mazzone

**Affiliations:** 1 Lab of Molecular Oncology and Angiogenesis, Department of Oncology, KU LeuvenLeuven, Belgium; 2 Lab of Molecular Oncology and Angiogenesis, Vesalius Research Center, VIBLeuven, Belgium; 3 Cancer Research UK Beatson InstituteGlasgow, UK; 4 Faculty of Biochemistry and Molecular Medicine and Biocenter Oulu, University of OuluOulu, Finland; 5 Department of Biochemistry, Center for Molecular Biomedicine, Institute for Biochemistry and Biophysics, Friedrich Schiller University of JenaJena, Germany; 6 Department of General, Visceral and Transplantation Surgery, University of HeidelbergHeidelberg, Germany; 7 Digestive Oncology Department, University Hospitals LeuvenLeuven, Belgium; 8 Oulu Center for Cell-Matrix Research, Biocenter Oulu and Faculty of Biochemistry and Molecular Medicine, University of OuluOulu, Finland

**Keywords:** chemotherapy resistance, colorectal cancer, DNA repair, prolyl hydroxylase domain proteins, tumor suppressor p53

## Abstract

Overcoming resistance to chemotherapy is a major challenge in colorectal cancer (CRC) treatment, especially since the underlying molecular mechanisms remain unclear. We show that silencing of the prolyl hydroxylase domain protein PHD1, but not PHD2 or PHD3, prevents p53 activation upon chemotherapy in different CRC cell lines, thereby inhibiting DNA repair and favoring cell death. Mechanistically, PHD1 activity reinforces p53 binding to p38α kinase in a hydroxylation-dependent manner. Following p53–p38α interaction and chemotherapeutic damage, p53 can be phosphorylated at serine 15 and thus activated. Active p53 allows nucleotide excision repair by interacting with the DNA helicase XPB, thereby protecting from chemotherapy-induced apoptosis. In accord with this observation, PHD1 knockdown greatly sensitizes CRC to 5-FU in mice. We propose that PHD1 is part of the resistance machinery in CRC, supporting rational drug design of PHD1-specific inhibitors and their use in combination with chemotherapy.

## Introduction

Resistance to chemotherapy remains a major clinical issue in the treatment of colorectal cancer (CRC). Response rates have already improved to about 30–40% over the past years with the introduction of the currently used FOLFOX and FOLFIRI regimens in patients with metastatic CRC, however still leaving room for further research on potential candidates causing chemorefractory disease (Prenen *et al*, 2013).

Prolyl hydroxylase domain proteins PHD1, PHD2, and PHD3 (codified by *EGLN2*, *EGLN1,* and *EGLN3*, respectively) are oxygen-sensitive enzymes initially known for their ability to target the hypoxia-inducible transcriptional factors HIF-1α and HIF-2α for proteasomal degradation (Epstein *et al*, 2001). Besides controlling cellular adaptation to hypoxic conditions, it is now clear that PHDs are also involved during cell damage and metabolic stress (Aragones *et al*, 2008; Schneider *et al*, 2010; Leite de Oliveira *et al*, 2012). Recently, we have shown that inhibition of PHD2 mounts an adaptive response in mice treated with chemotherapeutic drugs, resulting in the protection against their toxic side effects (Leite de Oliveira *et al*, 2012). Additionally, PHD1 or PHD2 inhibition confers organ protection against ischemic damage (Aragones *et al*, 2008; Schneider *et al*, 2010; Takeda *et al*, 2011). However, proteins other than HIF-1α and HIF-2α (including PKM2, FOXO3a, ATF4, RPB1, HCLK2, and β_2_-adrenergic receptor) have been proven to be alternative targets of PHD1–3 (Epstein *et al*, 2001; Mikhaylova *et al*, 2008; Chan *et al*, 2009; Xie *et al*, 2009, 2012; Xue *et al*, 2010; Ameln *et al*, 2011; Hiwatashi *et al*, 2011; Takeda *et al*, 2011; Scholz *et al*, 2013; Wong *et al*, 2013; Zheng *et al*, 2014). In some cases, substrate hydroxylation by PHD1–3 does not initiate a consensus for proteasomal degradation. For example, RPB1 is positively regulated by PHD1-mediated hydroxylation (Mikhaylova *et al*, 2008). This suggests that many PHD targets and functions are still undiscovered. This is also reflected by the lack of a conclusive role for PHDs in the context of cancer biology in general. More specifically in CRC, PHD2 and PHD3 have been suggested to act as tumor suppressors because of their decreased expression in the cancer compared to the normal tissue (Chan *et al*, 2009; Xue *et al*, 2010). However, the expression data on PHD1 in CRC are not unison with some studies reporting decreased expression of PHD1 and others showing no alterations (Jubb *et al*, 2009; Rawluszko *et al*, 2013). Importantly, none of these studies correlate the activity (rather than the expression) of these enzymes to disease onset and outcome or even, more specifically, to CRC response to chemotherapeutic regimens.

The transcription factor p53 is undoubtedly the most characterized cell stress sensor and tumor suppressor. p53 is usually phosphorylated and activated upon oncogene activation and DNA damage resulting in growth arrest and DNA repair or cell death induction, depending on the extent of the damage (Vousden & Lane, 2007). In CRC, p53 is mutated in about 50% of patients. However, p53 is never the primary hit and a clear correlation between p53 mutations and patient survival has never been proven (Tejpar *et al*, 2010), suggesting that p53 could potentially play both a sensitizing and desensitizing role against chemotherapy, depending on the cellular context as previously proposed (Ferreira *et al*, 1999). By using *in vitro* and *in vivo* mouse models, in this study, we investigate whether and how PHDs and p53 are intertwined and play a role in the resistance toward chemotherapy in colorectal cancer.

## Results

### *PHD1* silencing hinders p53 activation upon chemotherapy treatment

**Figure 1:**
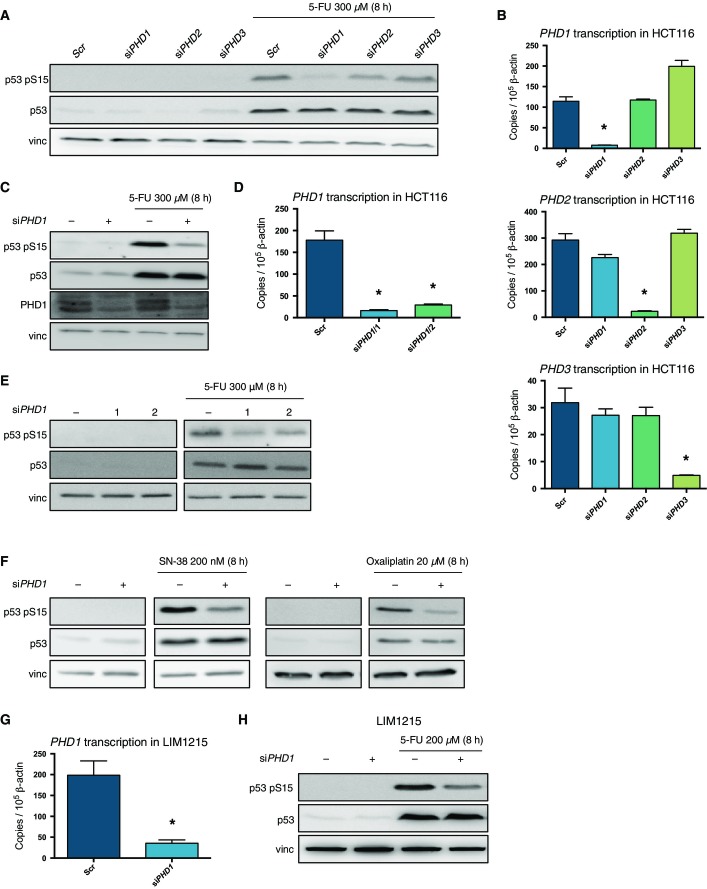
Silencing of *PHD1* decreases p53 phosphorylation in response to chemotherapy in CRC cells Western blot for p53, phosphorylated p53 at Ser15 (p53 pS15), and vinculin (vinc) in HCT116 treated with 300 μM 5-FU for 8 h and silenced for *PHD1*, *PHD2,* or *PHD3*.
qRT–PCR for *PHD1*, *PHD2,* and *PHD3* in HCT116 silenced for *PHD1* (*P = 0.0006 toward Scr), *PHD2* (*P = 0.0004 toward Scr), or *PHD3* (*P = 0.0075 toward Scr). A two-tailed unpaired *t*-test was performed with *n* = 3/group.
Western blot of p53 pS15, p53, PHD1, and vinculin in HCT116 treated with 300 μM 5-FU for 8 h and silenced for *PHD1* or a scrambled (Scr) control.
qRT–PCR for *PHD1* showing silencing efficacy with both siRNAs 1 (*P = 0.0017 toward Scr) and 2 (*P = 0.0023 toward Scr) compared to the scrambled control in HCT116. A two-tailed unpaired *t*-test was performed with *n* = 3/group.
Western blot for p53, p53 pS15, and vinc in HCT116 silenced for *PHD1* with two different constructs (constructs 1 and 2) upon exposure to 300 μM 5-FU for 8 h.
Western blot for p53 pS15, p53, and vinculin in HCT116 silenced for *PHD1* and treated with either 200 nM SN-38 or 20 μM oxaliplatin for 8 h.
RNA levels of *PHD1* in LIM1215 silenced for *PHD1*. (*P = 0.0099 toward Scr in a two-tailed unpaired *t*-test with *n* = 3/group.)
Western blot for p53 pS15, p53, and vinc in LIM1215 upon silencing of *PHD1* and treatment for 8 h with 200 μM 5-FU. Data information: Vinculin (vinc) was used as a loading control in (A, C, E, F, H). Source data are available online for this figure.

To evaluate the possible effect of PHD1–3 on p53 activation upon chemotherapy treatment, we silenced *EGLN2*, *EGLN1*, or *EGLN3* (coding for PHD1, PHD2, and PHD3*,* respectively) in *p53*^wt/wt^ HCT116 cells (also denoted HCT116) (Sur *et al*, 2009) and treated them with 5-FU. *PHD1*, *PHD2,* and *PHD3* RNA transcripts after knockdown were reduced, respectively, by 86.4, 91.1 and 84.7% compared to the scrambled control (Fig 1B). Evaluation of p53 activation was done by Western blotting for p53 phosphorylation at Ser15 (p53 pS15), frequently associated with the initial steps of p53 activation (Meek & Anderson, 2009). Indeed, upon 5-FU treatment, HCT116 showed an increased p53 accumulation and phosphorylation at Ser15 in the scrambled control cells (Fig 1A). Silencing of *PHD2* or *PHD3* did not affect either p53 levels or phosphorylation both at baseline and after 5-FU treatment. However, *PHD1* knockdown significantly reduced p53 phosphorylation at Ser15 upon 5-FU treatment comparing to the scrambled control (Fig 1A and C). Similar results were obtained by using a different siRNA against *PHD1* (Fig 1D and E).

To address whether the reduction in p53 phosphorylation upon *PHD1* silencing also holds true upon different chemotherapeutics clinically used in CRC treatment, we exposed HCT116 to either SN-38 or oxaliplatin. In scrambled control cells, both drugs induced p53 phosphorylation, which was largely prevented upon silencing of *PHD1* (Fig 1F). To extend our findings to different CRC cell lines other than HCT116, we used LIM1215 carrying wild-type p53 (Chen *et al*, 2014). *PHD1* mRNA levels were 82.1% reduced in *PHD1*-silenced cells comparing to their scrambled control (Fig 1G). Treatment with 5-FU resulted in increased p53 levels and increased p53 phosphorylation at Ser15 compared to untreated cells; *PHD1* silencing strongly prevented this induction (Fig 1H).

Altogether, these data provide evidence that, in the context of colorectal cancer, a drop in PHD1 levels reduces p53 phosphorylation following the administration of three different chemotherapeutics commonly used in the clinical treatment of CRC.

### *PHD1* silencing sensitizes colorectal cancer cells to chemotherapy

**Figure EV1:**
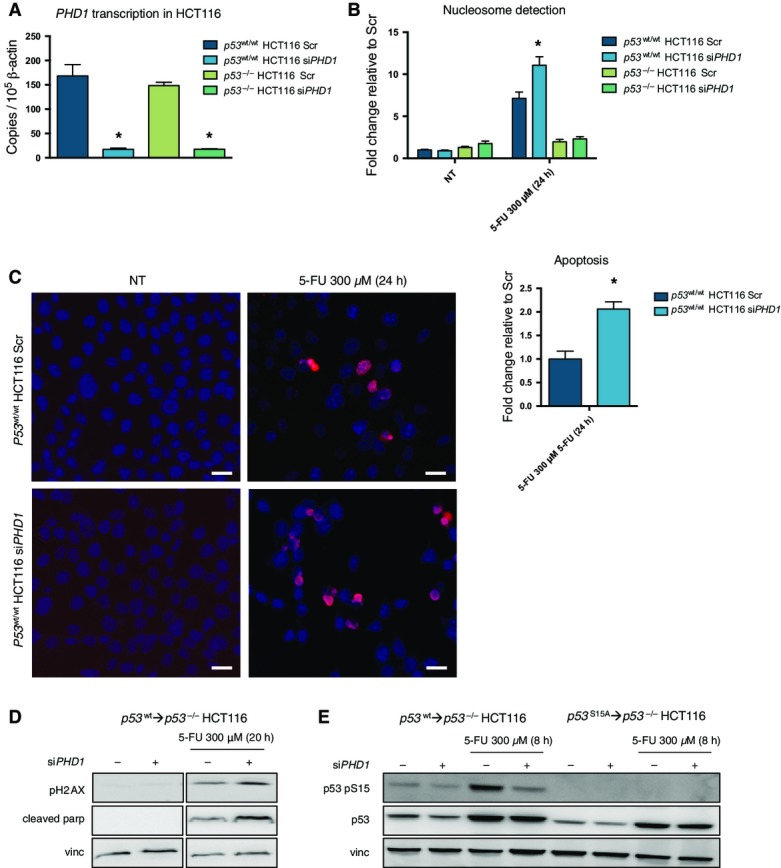
Silencing of *PHD1* increases cell apoptosis after chemotherapy RNA levels of *PHD1* in *p53*^wt/wt^ (*P = 0.0027 toward *p53*^wt/wt^ HCT116 Scr) and *p53*^−/−^ HCT116 (*P < 0.0001 toward *p53*^−/−^ HCT116 Scr) silenced for *PHD1*. A two-tailed unpaired *t*-test was performed with *n* = 3/group.
Nucleosome detection as a readout for apoptosis in *p53*^wt/wt^ and *p53*^−/−^ HCT116 silenced for *PHD1* after 24 h of 300-μM 5-FU treatment. *P = 0.04 toward the Scr 5-FU-treated condition as tested by a two-tailed unpaired *t*-test with *n* = 3/group.
Apoptosis in HCT116 silenced for *PHD1* and treated with 300 μM 5-FU for 24 h as detected by TUNEL immunocytochemistry and the quantification of these results. Scale bar represents 20 μm. *P = 0.003 toward the Scr 5-FU-treated condition as tested by a two-tailed unpaired *t*-test with *n* = 4/group.
Western blot detection of p53 pS15, p53, and vinculin (vinc) in *p53*^wt^→*p53*^−/−^ and *p53*^S15A^→*p53*^−/−^ HCT116 cells treated with 300 μM 5-FU for 8 h.
Parp cleavage, pH2AX, and vinc in *p53*^wt^→*p53*^−/−^ HCT116 cells upon 20-h treatment with 300 μM 5-FU. Data information: Vinc was used as a loading control in (D, E).

**Figure 2:**
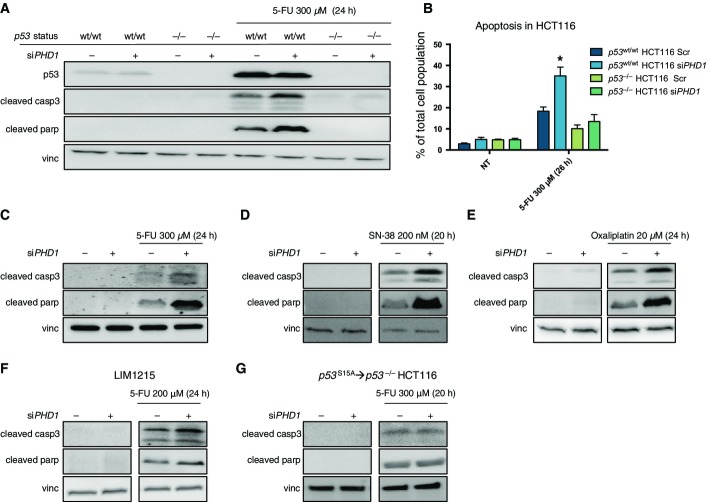
Silencing of *PHD1* increases cell apoptosis after chemotherapy A Western blot for p53, cleaved caspase-3 (cleaved casp3), cleaved parp, and vinculin (vinc) in *p53*^wt/wt^ and *p53*^−/−^ HCT116 silenced for *PHD1* upon exposure to 300 μM 5-FU for 24 h, showing an increased apoptotic response to 5-FU treatment in *PHD1*-silenced cells compared to the scrambled control.
B Confirmation of these results by FACS analysis of propidium iodide-stained *PHD1*-silenced *p53*^wt/wt^ and *p53*^−/−^ HCT116 cells exposed for 26 h to 300-μM 5-FU treatment. *P = 0.002 toward *p53*^wt/wt^ HCT116 shScr 5-FU-treated (two-tailed unpaired *t*-test) with *n* = 6 for non-treated *p53*^wt/wt^ HCT116 and *n* = 3 in all other groups.
C Detection of cleaved casp3, cleaved parp, and vinc in HCT116 silenced with a second siRNA for *PHD1*.
D, E Apoptosis as detected by Western blot for cleaved casp3 and parp in *PHD1*-silenced HCT116 treated for 20 h with 200 nM SN-38 (D) or for 24 h with 20 μM oxaliplatin (E).
F Western blot for cleaved casp3, parp, and vinc in LIM1215 silenced for *PHD1* and treated for 24 h with 200 μM 5-FU.
G Similar apoptosis levels were detected by Western blot for cleaved casp3 and parp with vinc as a loading control in *p53*^S15A^→*p53*^−/−^ HCT116 silenced for *PHD1* or scrambled (Scr) control and treated for 20 h with 300 μM 5-FU. Data information: Vinc is used as a loading control in (A, C–G). Source data are available online for this figure.

In order to find out whether the reduction in p53 phosphorylation after chemotherapy following *PHD1* knockdown could affect cell death in a p53-dependent manner, we treated *PHD1*-silenced *p53*^wt/wt^ HCT116 and the previously described *p53*^−/−^ HCT116 lacking full-length p53 (Sur *et al*, 2009) with 5-FU for 24 h (Fig EV1A). As expected, the treatment caused the cleavage of caspase-3 and parp in *p53*^wt/wt^ HCT116; however, this induction was further enhanced upon silencing of *PHD1* (Fig 2A). Though caspase-3 cleavage was also induced in *p53*^−/−^ HCT116 cells (albeit to a lower extent), it was not enhanced upon silencing of *PHD1*, suggesting that PHD1 might underlie resistance to chemotherapy by modulating p53 phosphorylation (Fig 2A). The same results were confirmed by FACS analysis on HCT116 cells stained with propidium iodide, by ELISA nucleosome detection and by TUNEL immunofluorescence staining of fixed cells (Figs 2B and EV1B and C). These findings were recapitulated by using a second siRNA against *PHD1* (Fig 2C). Similar to what was observed with 5-FU, treatments with either SN-38 or oxaliplatin were also able to promote apoptosis of *p53*^wt/wt^ HCT116 cells and this response was further enhanced by *PHD1* silencing (Fig 2D and E). To validate our observations in a different cell type, we showed that *PHD1* silencing was also able to sensitize *p53*^wt/wt^ LIM1215 cells to 5-FU (Fig 2F).

To link the effect of *PHD1* silencing on chemoresponse to the negative regulation of p53 phosphorylation at Ser15, we made use of *p53*^−/−^ HCT116 cells and reconstituted them with either wild-type p53 (*p53*^wt^→*p53*^−/−^ HCT116) or p53 mutated at Ser15 (*p53*^S15A^→*p53*^−/−^ HCT116). When measuring cell death, *p53*^wt^→*p53*^−/−^ HCT116 responded to 5-FU similar to their p53^wt/wt^ HCT116 counterpart, and correspondingly, *PHD1* silencing resulted in an increase in parp cleavage upon 5-FU treatment compared to control cells (Fig EV1D). In contrast, *p53*^S15A^→*p53*^−/−^ HCT116 no longer showed a differential apoptotic response in scrambled control and *PHD1*-silenced cells (Fig 2G). Consistently, *PHD1* silencing in *p53*^wt^→*p53*^−/−^ HCT116 cells reduced p53 Ser15 phosphorylation in response to 5-FU treatment, while p53 phosphorylation at Ser15 was not detected in *p53*^S15A^→*p53*^−/−^ HCT116 cells, confirming the specificity of the signal (Fig EV1E). Altogether, these data show that PHD1 inhibition can chemosensitize colorectal cancer cells by hindering p53 phosphorylation at Ser15.

### *PHD1* silencing improves the response of CRC to 5-FU treatment

**Figure 3:**
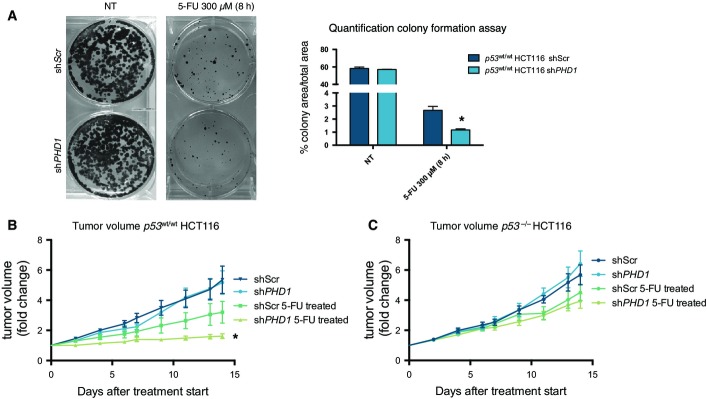
*PHD1* silencing sensitizes CRC to 5-FU treatment in mice Representative images and quantification of the colony formation capacity of *p53*^wt/wt^ HCT116 cells transduced with a doxycycline-inducible shScr or sh*PHD1* silencing construct, treated for 24 h with doxycycline with or without additional treatment for 8 h with 300 μM 5-FU. *P = 0.008 toward shScr, two-tailed unpaired *t*-test and *n* = 3/group.
Tumor volume of *p53*^wt/wt^ HCT116 cells transduced with a doxycycline-inducible shScr or sh*PHD1* silencing construct injected subcutaneously in nude mice and treated with 5-FU. *P = 0.045 toward shScr 5-FU-treated mice by two-way ANOVA with *n* = 6 for *p53*^wt/wt^ HCT116 shScr, *n* = 7 for *p53*^wt/wt^ HCT116 sh*PHD1* and *p53*^wt/wt^ HCT116 shScr 5-FU-treated, and *n* = 8 for *p53*^wt/wt^ HCT116 sh*PHD1* 5-FU-treated.
Tumor volume of *p53*^−/−^ HCT116 cells transduced with a doxycycline-inducible shScr or sh*PHD1* silencing construct injected subcutaneously in nude mice and treated with 5-FU. *n* = 6 for *p53*^−/−^ HCT116 shScr, *p53*^−/−^ HCT116 sh*PHD1,* and *p53*^−/−^ HCT116 shScr 5-FU-treated and *n* = 8 for *p53*^−/−^ HCT116 sh*PHD1* 5-FU-treated.

**Figure EV2:**
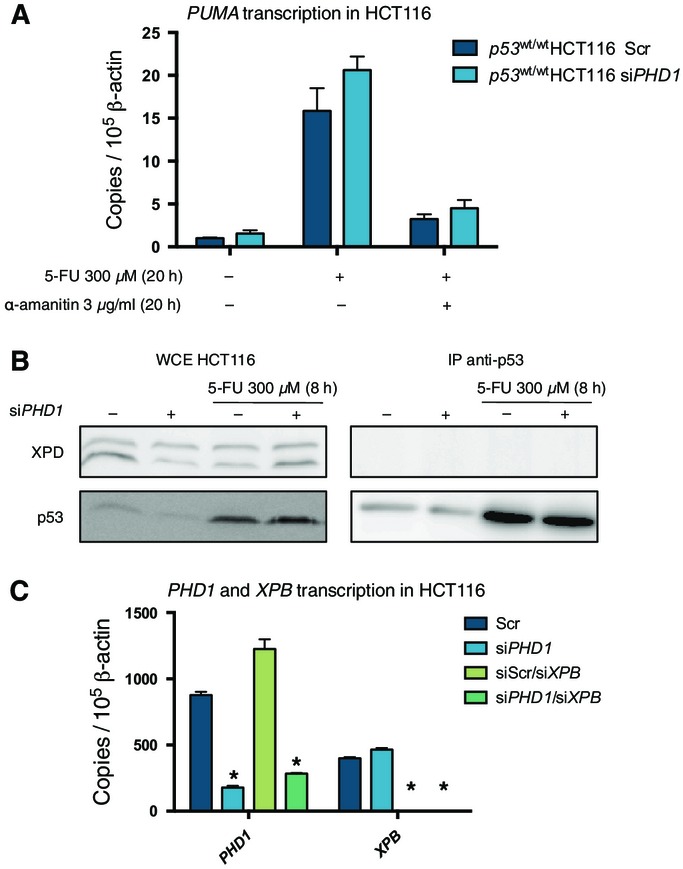
Colony formation with *p53*^−/−^ HCT116 cells is not altered by *PHD1* silencing Evaluation of *PHD1* transcription by qRT–PCR in *p53*^wt/wt^ (*P < 0.0001 toward *p53*^wt/wt^ HCT116 shScr) and *p53*^−/−^ HCT116 (*P < 0.0001 toward *p53*^−/−^ HCT116 shScr) silenced for *PHD1* by stable transduction with a doxycycline-inducible construct and treated with 1 μg/ml doxycycline for 24 h. A two-tailed unpaired *t*-test was performed with *n* = 6/group.
Colony formation and quantification in *p53*^−/−^ HCT116 shScr and sh*PHD1* after 8 h of 300 μM 5-FU, showing a similar decrease in colony formation between the two conditions compared to the non-treated cells.

To evaluate whether the aforementioned findings are also relevant in more complex systems, we initially performed a colony formation assay in *p53*^wt/wt^ HCT116 carrying a doxycycline-inducible shScr or sh*PHD1* construct. After treatment for 24 h with 1 μg/ml of doxycycline, cells were exposed to 5-FU in combination with doxycycline and then assessed for the ability to form foci *in vitro*. In the scrambled control, 5-FU treatment decreased colony formation compared to the untreated cells, but this difference was even further decreased upon silencing of *PHD1* (Figs 3A and EV2A). In contrast, no differences in colony formation capacity were detected between *PHD1*-silenced and control *p53*^−/−^ HCT116 cells upon their treatment with 5-FU (Fig EV2A and B), highlighting the dependency of p53 in the PHD1-mediated resistance against cytostatic drugs.

Following these results, we investigated the preclinical relevance of these findings *in vivo*. To this end, nude mice were sub-cutaneously injected with *p53*^wt/wt^ or *p53*^−/−^ HCT116, where conditional silencing of *PHD1* was achieved by doxycycline administration when tumors reached 250 mm^3^. Forty-eight hours after doxycycline administration, mice received a weekly treatment with the maximum tolerated dose of 100 mg/kg 5-FU. While tumor growth was not altered in untreated mice carrying *p53*^wt/wt^ HCT116 shScr or sh*PHD1* tumors, 5-FU treatment reduced tumor volume by 39.5% in *p53*^wt/wt^ HCT116 shScr, but by 70% in mice carrying a tumor silenced for *PHD1* (Fig 3B). In contrast, 5-FU-treated mice carrying *p53*^−/−^ HCT116 shScr or sh*PHD1* tumors did not show any differences in tumor growth, providing evidence for the p53 dependency of these findings (Fig 3C). These results show that *PHD1* silencing can increase the sensitivity of CRC to chemotherapeutic drugs both *in vitro* and *in vivo* in a p53-dependent manner.

### PHD1 hydroxylase promotes p53 phosphorylation upon chemotherapy

**Figure 4:**
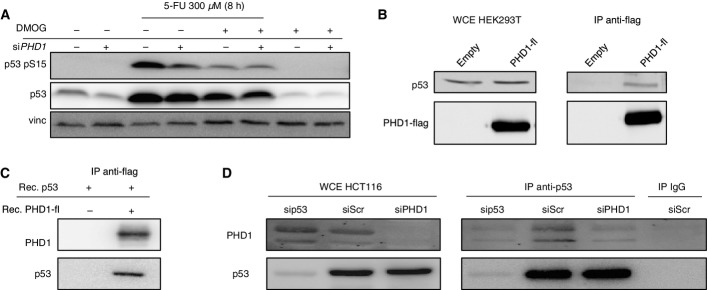
PHD1 hydroxylase is required for p53 phosphorylation upon chemotherapy treatment Western blot for p53 pS15, p53, and vinculin (vinc) in *PHD1*-silenced HCT116 upon treatment with 300 μM 5-FU for 8 h with or without 0.5 mM DMOG for 26 h. Vinculin (vinc) is used as a loading control.
Western blot detection of PHD1-Flag and p53 in whole cell extracts (WCE) or after immunoprecipitation against the Flag-tag of overexpressed PHD1-Flag in HEK293T cells.
Western blot detection of PHD1-Flag and p53 after immunoprecipitation against the Flag-tag of recombinant PHD1-Flag incubated with recombinant p53.
Western blot for p53 and PHD1 in WCE or after immunoprecipitation of endogenous p53 in *p53*^wt/wt^ HCT116 cells, silenced with a Scr control, si*PHD1,* or si*p53,* or after immunoprecipitation of *p53*^wt/wt^ HCT116 cell lysates with an IgG control. Source data are available online for this figure.

**Figure EV3:**
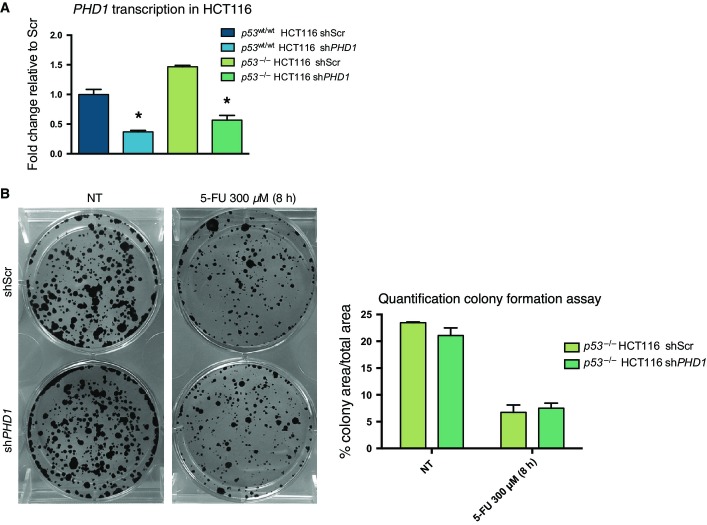
HIFs do not play a role in the regulation of p53 phosphorylation upon PHD1 silencing A Western blot detection of p53 pS15, p53, HIF-1α, and vinculin (vinc) in HCT116 silenced for *PHD1* either alone or in combination with *HIF-1α* or *HIF-2α* silencing.
B, C qRT–PCR for *HIF-1α* (B, *P = 0.0003 and ^#^*P* = 0.0003 both toward Scr) and *HIF-2α* (C, *P = 0.001 and ^#^*P* = 0.0007 both toward Scr) in HCT116 silenced for *PHD1* alone or in combination with either of the respective genes. A two-tailed unpaired *t*-test was performed with *n* = 3/group.

Mechanistically, PHDs have been shown to affect other proteins in both hydroxylation-dependent and hydroxylation-independent manners (Mikhaylova *et al*, 2008; Chan *et al*, 2009; Xue *et al*, 2010; Hiwatashi *et al*, 2011; Takeda *et al*, 2011; Zheng *et al*, 2014). To evaluate whether the enzymatic function of PHD1 was required for p53 regulation, scrambled and *PHD1*-silenced HCT116 cells were treated with the non-specific prolyl hydroxylase inhibitor DMOG in the presence or absence of 5-FU. DMOG treatment alone did not significantly affect p53 levels or phosphorylation at Ser15; however, it did decrease p53 phosphorylation upon 5-FU treatment in control cells, thus mimicking the effect of *PHD1* knockdown. *PHD1* silencing did not further reduce the phosphorylation of p53 (Fig 4A), providing evidence that PHD1 promotes p53 phosphorylation through its hydroxylase function. To exclude that HIFs could play a role in this process as they have been shown to influence p53 levels and activity (Sermeus & Michiels, 2011), we silenced *HIF-1α* or *HIF-2α* in combination with *PHD1* in HCT116. Upon treatment with 5-FU, silencing of *PHD1* in combination with *HIF-1*α or *HIF-2α* knockdown still impaired p53 Ser15 phosphorylation compared to the scrambled control (Fig  EV3A–C), indicating that PHD1-mediated p53 regulation is HIF independent.

To investigate whether PHD1 could interact with p53, Flag-tagged PHD1 was overexpressed in HEK293T cells and immunoprecipitated using anti-Flag antibodies. From this analysis, we observed that endogenous p53 was able to bind PHD1 (Fig 4B). Furthermore, to evaluate whether this interaction was direct or indirect, we immunoprecipitated recombinant Flag-tagged PHD1 in the presence of recombinant p53. Also in this experimental setting, PHD1 was able to pull down p53, proving a direct interaction of these two proteins (Fig 4C). We next wanted to see whether endogenous PHD1 interacts with endogenous p53 in HCT116 cells. To this end, we immunoprecipitated p53 from *p53*^wt/wt^ HCT116 cells and probed the precipitate with PHD1 and p53 antibodies. To control binding specificity, we also used *p53*^−/−^ or *PHD1-*silenced HCT116 cells and performed an IP with an unrelated IgG control antibody. The data obtained show that PHD1 could interact with p53 also at the endogenous level in HCT116 (Fig 4D).

In summary, we can conclude that PHD1 through its hydroxylation function and binding with p53 allows p53 phosphorylation at Ser15 upon chemotherapy treatment.

### Reduced p53 hydroxylation impairs p38α-mediated p53 phosphorylation

**Figure EV4:**
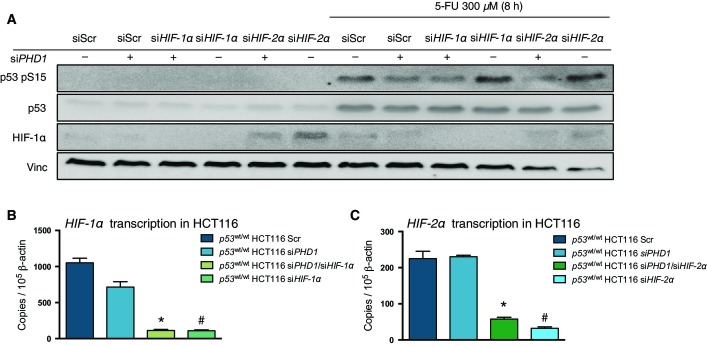
Silencing efficiency in HCT116 cells Western blot for p53 pS15, p53, and vinculin (vinc) in HCT116 silenced for either *PHD1*, *Chk1*, *Chk2*, *ATR*, *ATM*, *DNA-PK,* and *p38α* and treated with 300 μM 5-FU for 8 h.
Analysis by qRT–PCR of *PHD1* (*P = 0.01 toward Scr), *Chk1* (*P = 0.007 toward Scr), *Chk2* (*P = 0.002 toward Scr), *ATR* (*P = 0.002 toward Scr), *ATM* (*P = 0.002 toward Scr), *DNA-PK* (*P = 0.001 toward Scr), and *p38α* (*P = 0.0001 toward Scr) transcription in HCT116 upon their respective silencing in comparison with the Scr control. A two-tailed unpaired *t*-test was performed with *n* = 3/group.
Analysis by qRT–PCR of *PHD1* (*P < 0.0001 and ^#^*P* = 0.0009 both toward Scr by a two-tailed unpaired *t*-test with *n* = 6 for Scr and si*PHD1* and *n* = 3 for siPHD1/si*p38α* and si*p38α*) and *p38α* (*P = 0.006 and ^#^*P* = 0.02 both toward Scr by a two-tailed unpaired *t*-test with *n* = 3/group) upon silencing of these genes in comparison with Scr in HCT116.

**Figure 5:**
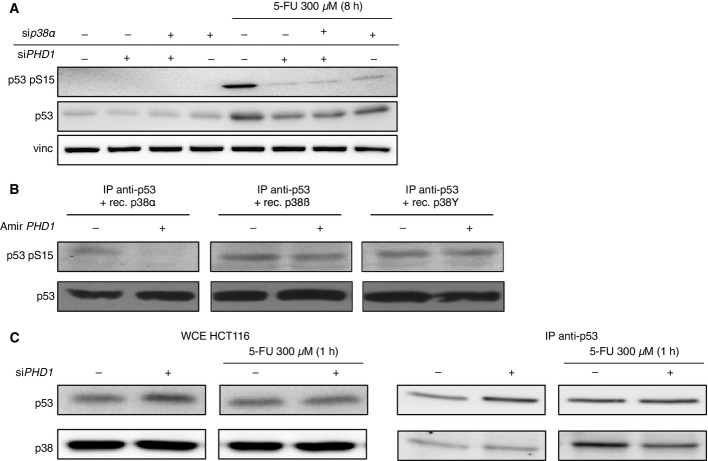
PHD1 hydroxylase favors p38α-mediated p53 phosphorylation Western blot for p53 pS15, p53, and vinculin (vinc) in HCT116 treated with 300 μM 5-FU upon silencing of *PHD1* and *p38α*, alone or in combination. Vinculin (vinc) is used as a loading control.
Detection of p53 pS15 and p53 after *in vitro* phosphorylation by p38α, p38β, or p38γ of p53 immunoprecipitated from cells silenced for a control (SIMA) or *PHD1*.
Detection by Western blot of p53 and p38 from whole cell extracts (WCE) or after immunoprecipitation of p53 from cell silenced for a Scr control or si*PHD1* and treated for 1 h with 300 μM 5-FU. Source data are available online for this figure.

To study how *PHD1* silencing could affect p53 phosphorylation at Ser15, we first assessed which of the known p53-kinases were involved in p53 phosphorylation at Ser15. Therefore, we silenced *Chk1*, *Chk2*, *ATR*, *ATM*, *p38α*, and *DNA-PK* in HCT116 cells upon treatment with 5-FU (Bode & Dong, 2004; Meek & Anderson, 2009) (Fig EV4A and B). Unexpectedly, at least under the experimental conditions tested, only *p38α* silencing could completely reduce p53 phosphorylation at Ser15 to a similar extent as silencing of *PHD1* (Fig EV4A). To confirm whether PHD1 could indeed affect p53 phosphorylation through p38α, we silenced *PHD1* and *p38α*, alone or in combination (Figs 5A and Fig EV4C). After treatment with 5-FU, silencing of both *p38α* and *PHD1* did not show an additive reduction in p53 phosphorylation at Ser15, therefore suggesting that PHD1 hydroxylase function allows proper p53 phosphorylation by p38α (Fig 5A). To further confirm the specificity of these findings for p38α, we performed an *in vitro* kinase assay with p38α, p38β, and p38γ on p53 isolated from cells expressing either a control (mirSIMA) or an artificial miRNA against *PHD1* (mir*PHD1*). This revealed that only phosphorylation of p53 by p38α, but not by p38β or p38γ, was prevented when PHD1 was lacking (Fig 5B). Finally, we proved that, upon 5-FU treatment, *PHD1* silencing significantly reduced p53 binding to p38, confirming that hydroxylation of p53 is required for p38 interaction to p53 (Fig 5C). These data show that lack of PHD1 prevents proper p53 phosphorylation through a reduced binding of p53 to p38α upon chemotherapy treatment.

### *PHD1* silencing reduces p53-mediated DNA repair following chemotherapy

**Figure 6:**
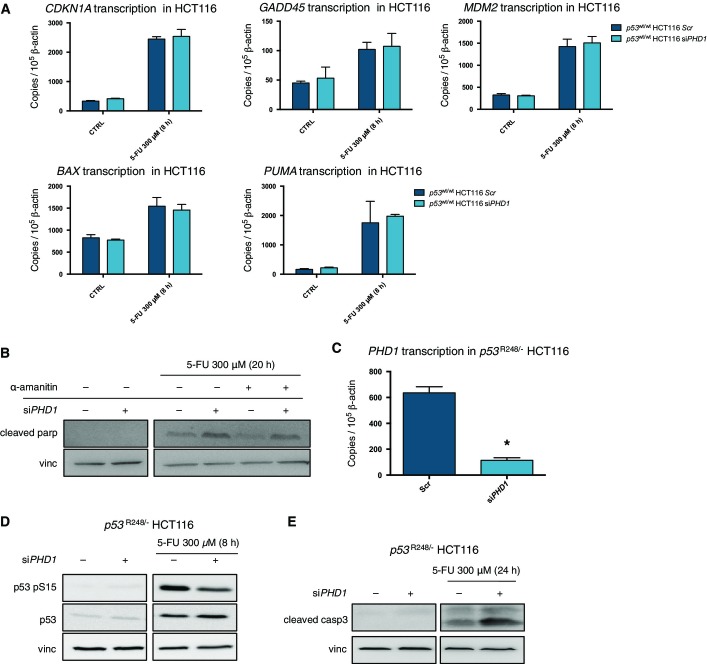
*PHD1* silencing increases apoptosis in a transcription-independent manner Transcription analysis of *CDKN1A*, *GADD45*, *MDM2*, *BAX*, *PUMA* in *PHD1*-silenced *p53*^wt/wt^ HCT116 either untreated or treated for 8 h with 300 μM 5-FU.
Western blot for cleaved parp and vinculin (vinc) in *PHD1*-silenced HCT116 treated for 20 h with 300 μM 5-FU alone or in combination with 3 μg/ml α-amanitin.
*PHD1* mRNA levels in *p53*^R248/−^ HCT116 silenced for *PHD1*. *P = 0.0005 toward scrambled control with a two-tailed unpaired *t*-test and *n* = 3/group.
Western blot for p53 pS15, p53, and vinculin in *PHD1*-silenced *p53*^R248/−^ HCT116 upon 8-h treatment with 300 μM 5-FU.
Western blot for cleaved casp3 and vinculin in *p53*^R248/−^ HCT116 silenced for *PHD1* and treated with 300 μM 5-FU for 24 h. Data information: Vinc was used as a loading control in (B, D, E). Mean values ± s.e.m. Source data are available online for this figure.

**Figure EV5:**
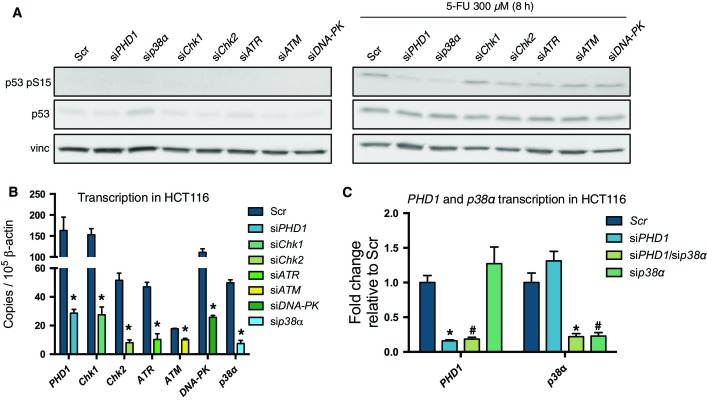
Transcription-independent regulation of p53 by PHD1 Transcriptional analysis of *PUMA* in HCT116 shows that addition of 3 μg/ml α-amanitin can block the induction of *PUMA* after 20 h of 300 μM 5-FU treatment.
Immunoprecipitation of p53 in *PHD1*-silenced HCT116 which were untreated or treated with 300 μM 5-FU for 8 h, showing no interaction between p53 and XPD.
Analysis by qRT–PCR of *PHD1* and *XPB* upon silencing of these genes in comparison with Scr in HCT116. *P < 0.0001 compared to the Scr control by a two-tailed unpaired *t*-test with *n* = 3/group.

To investigate how the silencing of *PHD1* could result in an increased apoptotic response, we initially focused our attention on the transcription of several important target genes of p53 such as *CDKN1A*, *GADD45*, *MDM2*, *BAX,* and *PUMA* (Menendez *et al*, 2009). Although all these genes were induced by 5-FU treatment in the scrambled control condition, *PHD1* silencing did not affect their transcription compared to the control both at baseline and upon 5-FU treatment (Fig 6A). Therefore, we evaluated whether transcriptional activity was at all required for this specific process by treating HCT116 cells with 5-FU together with the transcription inhibitor α-amanitin. The proper function of the compound was confirmed by quantification of *PUMA* transcription, a downstream target of p53 (Fig Fig EV5A). Silencing of *PHD1* was still able to increase parp cleavage upon combined treatment with 5-FU and α-amanitin. Thus suggesting that p53-mediated transcription is not the cause for the increased cell death observed after chemotherapy upon *PHD1* silencing (Fig 6B). In line with this, we also evaluated apoptosis after 5-FU treatment in *p53*^R248/−^ HCT116, carrying a p53 DNA contact mutant, incapable of DNA binding and transcription despite a well-preserved tridimensional structure (Muller & Vousden, 2013). As already suggested by the use of α-amanitin, also in these cells we noted that silencing of *PHD1* upon 5-FU treatment could still reduce Ser15 phosphorylation (Fig 6C and D) and results in increased apoptosis as detected by Western blot for cleaved caspase-3 (Fig 6E). These experiments confirm the importance of a transcription-independent function of p53.

**Figure 7:**
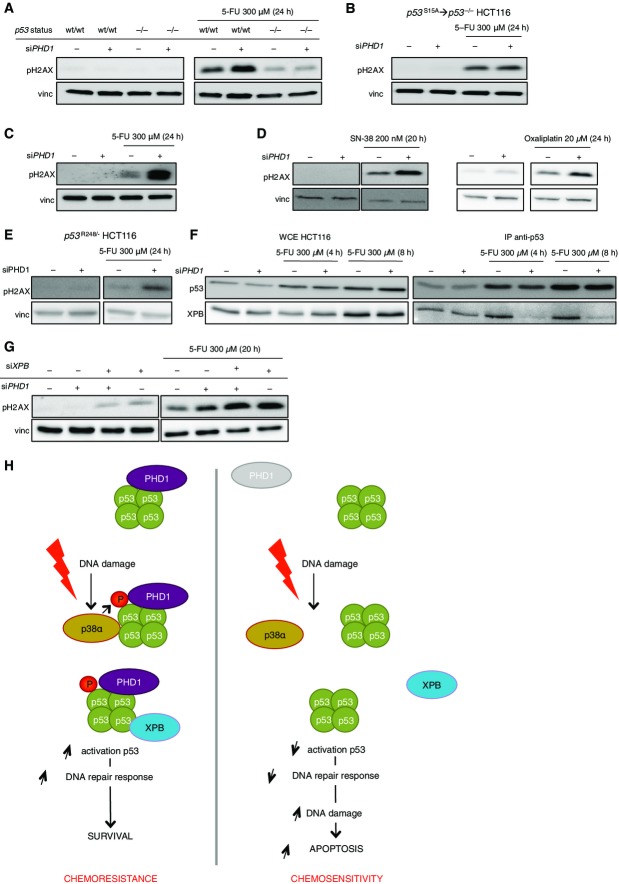
*PHD1* silencing impairs p53-mediated DNA repair by reducing p53 binding to the NER helicase XPB Western blot for pH2AX and vinculin (vinc) in *p53*^wt/wt^ and *p53*^−/−^ HCT116 cells after 24-h treatment with 300 μM 5-FU.
Detection of pH2AX and vinc in *p53*^S15A^→*p53*^−/−^ HCT116 cells silenced for *PHD1* and treated with 300 μM 5-FU for 24 h.
Detection of pH2AX and vinc in HCT116 silenced with a second silencing construct of *PHD1* and treated for 24 h with 300 μM 5-FU.
Western blot for pH2AX and vinc in HCT116 treated for 20 h with 200 nM SN-38 or 24 h with 20 μM oxaliplatin.
Western blot for pH2AX and vinc in *p53*^R248/−^ HCT116 cells silenced for *PHD1* upon treatment with 300 μM 5-FU for 24 h.
Detection by Western blot of p53 and XPB from whole cell extracts (WCE) or after immunoprecipitation of p53 from cells silenced for Scr control or si*PHD1* and treated for 4 or 8 h with 300 μM 5-FU.
Western blot for pH2AX and vinc in HCT116 silenced for *PHD1* alone or in combination with silencing for *XPB* upon treatment with 300 μM 5-FU for 20 h.
PHD1 hydroxylase activity allows the phosphorylation of p53 upon chemotherapy treatment in colorectal cancer cell lines. As a consequence, p53 can interact with the NER machinery and more specifically with XPB. This promotes DNA repair and results in the resistance toward the cytostatic effects of chemotherapy (left panel). On the other hand, when PHD1 is lacking, the p53-kinase p38α can no longer bind, and therefore, p53 phosphorylation upon chemotherapy treatment is prevented. In absence of Ser15 phosphorylation, p53 is no longer able to bind XPB, thereby blocking the DNA repair-promoting capacity of p53. This leads to increased DNA damage and thus enhanced apoptotic response to chemotherapy (right panel). Data information: Vinc is used as a loading control in (A–E, G). Source data are available online for this figure.

As p53 has been shown to influence DNA repair in a transcription-independent manner, and as DNA repair can increase the resistance of cancer cells toward chemotherapy (Bouwman & Jonkers, 2012; Gordon & Nelson, 2012), we evaluated the DNA damage present in the cells. Indeed upon 5-FU treatment, DNA damage, detected by phosphorylation of histone H2AX (pH2AX) as a marker, was induced in the scrambled control and this was further increased in *PHD1*-silenced cells (Fig 7A). The increased DNA damage consequent to *PHD1* silencing after chemotherapy treatment was p53 dependent as pH2AX accumulation in *p53*^−/−^ HCT116 upon 5-FU treatment was equal in either the presence or absence of PHD1 (Fig 7A). More specifically, the reduced phosphorylation of p53 at Ser15 is required for the increased DNA damage, as there are no longer differences between the Scr control and si*PHD1* condition in pH2AX accumulation upon 5-FU treatment in the *p53*^S15A^→*p53*^−/−^ HCT116 cells (Figs 7B and EV1D). These results were also confirmed with a second siRNA against *PHD1* (Fig 7C) as well as upon SN-38 or oxaliplatin treatment (Fig 7D) and in the *p53*^R248/−^ HCT116 cells (Fig 7E).

Previous studies have shown that p53 can stimulate the nucleotide excision repair (NER) pathway (engaged after 5-FU-, SN-38-, and oxaliplatin-induced DNA damage) in a transcription-independent manner through its direct interaction with XPB and XPD, two components of transcription factor II human (TFIIH) (Sengupta & Harris, 2005). When assessing the physical interaction of p53 with XPB or XPD, we noticed that *PHD1* knockdown strongly reduced the binding of p53 and XPB upon treatment with 5-FU (Fig 7F), whereas no interaction between XPD and p53 was detected (Fig Fig EV5B). To confirm that indeed, these findings are dependent on XPB, we silenced *PHD1* either alone or in combination with *XPB* in HCT116 treated with 5-FU (Fig EV5C). The silencing of *XPB* caused a strong induction of DNA damage as detected by pH2AX upon 5-FU treatment and this was not further enhanced upon additional silencing of *PHD1* (Fig 7G). These data show that impaired binding of p53 to XPB is indeed the underlying cause of the reduced DNA repair capacity of the cells after PHD1 blockade. In essence, the reduced p53 phosphorylation at Ser15 upon *PHD1* silencing reduces p53 binding to XPB in the NER complex upon chemotherapeutic challenge, thereby increasing DNA damage and consequently cell death upon cytostatic insult (Fig 7H).

## Discussion

Chemotherapy remains the most widely used cancer treatment. In the past, much attention has been paid on the mechanisms underlying chemotherapy resistance. In our study, we aimed to investigate the interplay between PHDs and p53 and their potential role in the response of CRC to chemotherapy. The model we propose relies on PHD1 hydroxylase function allowing p38α-dependent phosphorylation of p53 in response to cytostatic damage in colorectal cancer cell lines. As a consequence, p53 can interact with the NER machinery and more specifically with XPB. This promotes DNA repair and results in the resistance to chemotherapy (Fig 7H, left panel). On the other hand, when PHD1 is inhibited, the p53-kinase p38α can no longer bind, and therefore, p53 phosphorylation upon chemotherapy treatment is prevented. It follows that p53 is not any longer able to bind XPB, thereby impairing the DNA repair capacity linked to p53 activity. This leads to increased DNA damage and cell death in response to the chemotherapeutic treatment (Fig 7H, right panel).

With these findings, we are adding another layer of complexity to the role of PHD1 in cancer. Previous reports have shown that murine PHD1, when overexpressed in colorectal cancer cells, can decrease tumor growth through the reduction of HIF-1α and VEGF (Erez *et al*, 2003). On the other hand, a reduction in PHD1 levels in breast cancer can hinder tumor growth due to the accumulation of FOXO3a and consequent suppression of cyclin D1 eventually leading to a decreased proliferation (Zhang *et al*, 2009; Zheng *et al*, 2014). In non-cancerous cells, the situation is even more complex with increased HIF-2α and MYC-dependent proliferation in liver tissue of *Phd1* KO mice after liver resection (Mollenhauer *et al*, 2012), but decreased proliferation upon *PHD1* silencing in HeLa ovarian cells because of reduced hydroxylation of the centrosome component Cep192 (Moser *et al*, 2013) or eventually decreased enterocyte apoptosis in *Phd1* knockout mice affected by colitis (Tambuwala *et al*, 2010). In our current study, we uncover that *PHD1* silencing does not affect the colony formation capacity and tumor growth under basal conditions, but it increases sensitivity of CRC cells toward the cytostatic effects of chemotherapeutic drugs such as 5-FU, SN-38, or oxaliplatin *in vitro* and *in vivo*. These effects are independent of HIF signaling as proven by: (i) direct interaction of PHD1 and p53, (ii) PHD1-mediated p53 regulation, (iii) the absence of detectable HIF-2α levels, (iv) the lack of modulation of HIF-1α by *PHD1* silencing, and (v) reduced phosphorylation at Ser15 of p53 upon 5-FU treatment after silencing of *PHD1* even in the absence of HIF-1α or HIF-2α. Together with our experiments in *p53*^−/−^ HCT116 cells supporting the p53 dependency of the effects on DNA damage, apoptosis, and colony formation upon PHD1 regulation, these findings exclude the possibility that the previously shown interplay between HIFs and p53 (Sermeus & Michiels, 2011) can take part in the mechanism here described.

With our work, we show that PHD1 binds to p53 and that its hydroxylase function is required for the effects observed on p53 phosphorylation. This would suggest that indeed, p53 could be hydroxylated. The direct binding of p53 to PHD1 observed *in vitro* supports this hypothesis, as no other intermediate proteins are required for the interaction. Hereby, prolyl hydroxylation would represent a new posttranslational modification in the p53 field that adds to the long list of already known posttranslational p53 modifications (Bode & Dong, 2004). So far it was believed that the phosphorylation of p53 at Ser15 was one of the initial steps of p53 activation (Bode & Dong, 2004; Meek & Anderson, 2009). Our data would however suggest that binding of PHD1 to p53 and potential hydroxylation of p53 precede its phosphorylation adding specificity to the phosphorylation process of p53 and directing p53 activity toward DNA repair and cytostatic signaling.

When looking further downstream in the p53 pathway, our results suggest that a reduction in p53 phosphorylation at Ser15 without altering the total p53 levels affects specifically the transcription-independent DNA repair pathway of p53. Indeed, transcription of some important p53 target genes such as *CDKN1A*, *MDM2,* and *PUMA* is not altered upon *PHD1* silencing after 5-FU treatment. This is in line with some previous evidence showing that p53 phosphorylation is not the main determinant of p53 transcriptional activity as p53 transcription can also be triggered by mere p53 stabilization without phosphorylation (Stommel & Wahl, 2004; Kruse & Gu, 2009). This could explain the lack in transcriptional differences observed in our model, but also uncovers a new role for p53 phosphorylation at Ser15 in NER. Previous findings have shown that p53 can positively influence NER and that p53 can do this independently of transcription through the binding with XPB and XPD (Sengupta & Harris, 2005; Chang *et al*, 2008). In our results, we did not observe binding between p53 and XPD, but observed a robust binding between XPB and p53 after 5-FU treatment, which was strongly reduced upon *PHD1* silencing and thus upon reduced p53 phosphorylation at Ser15, which is followed by impaired DNA repair and resultant apoptosis. Despite that NER has already been involved in resistance to chemotherapy (Dabholkar *et al*, 2000; Reed *et al*, 2003; Bohanes *et al*, 2011), to our knowledge, this is the first report showing that p53 phosphorylation can affect the DNA repair machinery, though further investigations will be required to explain how posttranslational modifications in p53 can elicit this effect.

Here, we also show that mutation of p53 in position R248, a hot spot mutation in CRC, recapitulates the effects seen with wild-type p53. So far, mutations in p53 have been considered to be inactivating and rendering p53 incapable to perform any of its regular functions (Muller & Vousden, 2013). However, our data clearly demonstrate that a p53 DNA contact mutant is still regulated by PHD1 and retains at least a partial DNA-repair-promoting activity. Therefore, potential blockage of PHD1 could also be clinically relevant in patients carrying DNA contact mutations in p53—which are frequently reported in CRC.

The clinical usage of PHD1-specific inhibitors has been already proposed in the context of breast cancer, ischemic liver disease, and colitis (Zhang *et al*, 2009; Schneider *et al*, 2010; Tambuwala *et al*, 2010). Particularly in CRC, specificity of PHD inhibitors is warranted because PHD2 inhibition has been shown to increase tumor growth and PHD3 blockage has been associated with a reduced apoptotic response to irradiation (Chan *et al*, 2009; Xie *et al*, 2012). With our data, we pave the opportunity to design and validate PHD1-specific inhibitors in colorectal cancer patients carrying wild-type or DNA contact mutant p53 aiming to increase their sensitivity to currently used chemotherapeutic treatments.

## Materials and Methods

### Cell culture

HEK293T human embryonic kidney cells, HCT116 human colon carcinoma cells (containing wild-type p53: *p53*^wt/wt^ HCT116, further noted as HCT116; lacking p53: *p53*^−/−^ HCT116 and *p53*^R248/−^ HCT116, a kind gift from Prof. Karen Vousden, Glasgow), and the human colon cancer cell line LIM1215 (a kind gift from Prof. Sabine Tejpar, Leuven) were maintained at 37°C at 95% air and 5% CO_2_ in DMEM (Gibco, life technologies) supplemented with 10% fetal bovine serum (FBS, Gibco), 2 mM l-glutamine (Gibco), and 1% penicillin/streptomycin (PenStrep, Gibco). All cancer cell lines underwent mycoplasma testing before their use. Negative mycoplasma contamination status was verified using LookOut Mycoplasma PCR Kit (Sigma) and MycoAlert Mycoplasma Detection Kit plus Assay Control (Lonza). All cell lines were not maintained longer than 10 passages in culture to perform experimental procedures. Transfection of siRNA was performed with lipofectamine RNAiMAX (Life Technologies) according to the supplier’s protocol. Catalog numbers for the different siRNAs used can be found in Table 1.

**Table 1:** Sequences or ordering information of the silencing constructs used

si/shRNA	Reference
scramble	IDT: NC-1
si*PHD1*	IDT: HSC.RNAI.N053046.12.2
	HSC.RNAI.N053046.12.1
si*PHD2*	Ambion (Life Technologies): s99984
si*PHD3*	Ambion (Life Technologies): s41320
si*ATM*	IDT: HSC.RNAI.N000051.12.1
si*ATR*	IDT: HSC.RNAI.N001184.12.1
si*Chk1*	IDT: HSC.RNAI.N001114.12.1
si*Chk2*	IDT: HSC.RNAI.N00719412.2
si*p38α*	Ambion (Life Technologies):
	Hss 102353
	Hss 102352
si*DNA-PK*	IDT: HSC.RNAI.N001081640.12.1
si*HIF-1α*	IDT: HSC.RNAI.N001530.12.3
si*HIF-2α*	IDT: HSC.RNAI.N001430.12.3
si*XPB*	IDT: HSC.RNAI.N000122.12.1
mirSIMA	A kind gift from Prof. Acker (University of Giessen)
	Top sequence: TGCTGCATGAATATCTCTGTCTCCTTGTTTTGGCCACTGACTGACAAGGAGACAG ATATTCATG
	Bottom sequence: CCTGCATGAATATCTGTCTCCTTGTCAGTCAGTGGCCAAAACAAGGAGACAGAGA TATTCATGC
mir*PHD1*	A kind gift from Prof. Acker (University of Giessen)
	Top sequence: TGCTGGATGCTAGCTGATACTTGTCTGTTTTGGCCACTGACTGACAGACAAGTCAGCTAGCATC
	Bottom sequence: CCTGGATGCTAGCTGACTTGTCTGTCAGTCAGTGGCCAAAACAGACAAGTATCAGCTAGCATCC
pTripz-shScr	RHS4750 (GE Healthcare)
pTripz-sh*PHD1*	V3THS_377151 (GE Healthcare)

### Cell treatments

The chemotherapeutics 5-fluorouracil (5-FU, Sigma-Aldrich), 7-ethyl-10-hydroxycamptothecin (SN-38, Sigma-Aldrich), oxaliplatin (Selleck Chemicals), MG-132 (Calbiochem) as well as the PHD inhibitor dimethyloxaloylglycine (DMOG, Frontier Scientific) were prepared in DMSO stock solutions and diluted to their final concentration in DMEM (10% FBS, 2 mM l-glutamine, and 1% PenStrep). Treatment times and doses were used as indicated in the results.

### Western blot

Protein extraction was performed using RIPA buffer [50 mM Tris pH 8, 150 mM NaCl, 1% Triton X-100, 0.5% sodium deoxycholate, and 0.1% sodium dodecyl sulfate (SDS)] containing phosphatase (PhosSTOP Roche) and protease (Complete Roche) inhibitors. When the detection of apoptosis was required, the supernatants of the cells were also collected for Western blot analysis. The following antibodies were used for the detection of the proteins by immunoblotting: rabbit anti-PHD1 (Novus, NB100-310, 1/250 in 5% milk), mouse anti-p53 (Santa Cruz Biotechnology, DO1, 1/4,000 in 5% milk), rabbit anti-p53 (Santa Cruz Biotechnology, FL-393, 1/1,000 in 5% milk), mouse anti-vinculin (Sigma-Aldrich, V9131, 1/5,000 in 5% milk), rabbit anti-caspase-3 (Cell Signaling, #9665, 1/500 in 5% BSA), rabbit anti-Flag (Sigma-Aldrich, F7425, 1/1,000 in 5% milk), rabbit anti-phospho-serine 15 p53 (Cell Signaling, #9284, 1/1,000 in 5% BSA), rabbit anti-pH2AX (Cell Signaling, #2577, 1/1,000 in 5% BSA), rabbit anti-cleaved parp (Cell Signaling, #5625, 1/1,000 in 5% BSA), rabbit anti-p38 (Cell Signaling, #9212, 1/1,000 in 5% BSA), and rabbit anti-XPB (Santa Cruz Biotechnology, sc-293, 1/500 in 5% milk). Secondary goat anti-mouse and goat anti-rabbit antibodies directly conjugated to horseradish peroxidase were used (Santa Cruz Biotechnology, 1/4,000 in 5% milk), and blots were developed using ECL (Life Technologies) or Super Signal West Femto (Thermo Scientific) with a CCD camera (ImageQuantTM LAS 4000). All Western blot experiments were repeated at least three times in independent experiments, and uncropped figures can be found in the Source Data.

### Co-immunoprecipitation

Co-immunoprecipitation of PHD-Flagged proteins in HEK293T cells was performed by lysing the cells in RIPA buffer. After pre-clearing of 1 mg of protein lysate with 30 μl of non-conjugated Sepharose beads (GE Healthcare life sciences), 30 μl of ANTI-Flag® M2 Affinity Gel (Sigma-Aldrich, pre-blocked with 0.05% BSA) was added to the pre-cleared lysates. After a 2-h incubation at 4°C, the bead–sample complexes were washed three times with RIPA buffer. Afterward, 20 μg of Flag peptide (Sigma-Aldrich) diluted in 80 μl of TBS buffer [50 mM Tris–HCl pH 7.4, 150 mM NaCl, containing protease inhibitors (Complete Roche)] was added to the beads and incubated for 45 min at 4°C. Consequently, the eluted proteins were analyzed by immunoblot as described above.

Recombinant Flag-tagged PHD proteins were produced in Sf9 insect cells and were kindly provided by prof. Johanna Myllyharju (University of Oulu). Two milliliters of the insect cell pellet were homogenized in 200 μl of lysis buffer [150 mM NaCl, 100 mM glycine, 0.1% Triton X-100, 10 μM DTT, 10 mM Tris pH 7.8, 5 μM FeSO_4_, containing protease inhibitors (Complete Roche)] with 30 strokes in a glass Teflon homogenizer, while keeping on ice. The homogenates were centrifuged for 15 min at 14,707 *g*. The supernatant was filtered using a 0.45-μm filter and 20 μl ANTI-Flag® M2 Affinity Gel (Sigma-Aldrich) was added. After a 1-h incubation on a rotating wheel at 4°C, protein–bead complexes were washed three times in TBS [50 mM Tris–HCl pH 7.4, 150 mM NaCl, 5 μM FeSO_4_, containing protease inhibitors (Complete Roche)] and 1.24 μg recombinant p53 (a kind gift from Prof. Alan Fersht, University of Cambridge) was added. After a 1-h incubation on a rotating wheel at 4°C, beads were washed for three times with TBS buffer and 20 μg of Flag peptide (Sigma-Aldrich) diluted in 80 μl of TBS buffer was added to elute protein complexes from the beads by rotating for 1 h at 4°C. A small fraction of the eluate was then loaded on homemade polyacrylamide gels for Western blot detection.

Endogenous immunoprecipitation of the p53–PHD1 interaction was performed in HCT116 cells silenced for *PHD1* or *p53* or a scrambled control. Immunoprecipitation was performed with protein G dynabeads (Life Technologies) crosslinked to mouse anti-p53 (86 ng Santa Cruz DOI, 86 ng Santa Cruz BPp53-12, and 13 ng Merck Millipore OP03/μl beads) or mouse IgG control (185 ng Santa Cruz/μl beads) antibodies with BS_3_ (Life Technologies) according to the manufacturers’ protocol. Cells were then lysed in Saito’s modified lysis buffer [50 mM Tris–HCl pH 7.4, 0.15 M NaCl, 0.5% Triton X-100, 5 mM EDTA containing phosphatase (PhosSTOP Roche) and protease (Complete Roche)] and 2.5 mg of protein was added to 15 μl of mouse anti-p53- or mouse IgG-crosslinked dynabeads. The lysates were incubated for 2 h rotating at 4°C, after which they were washed 3 times with Saito’s modified lysis buffer, eluted by boiling 10 min in 40 μl 1× LDS buffer (Life Technologies). Supernatants were collected, DTT was added to a final concentration of 20 mM, and the samples were further boiled for 10 min after which they were analyzed by immunoblot as described above with the following antibodies: 1/3,000 mouse anti-p53 (DOI Santa Cruz) in 5% milk and 1/200 sheep anti-PHD1 (200 μg/ml R&D Systems AF6394) in 5% BSA.

### Quantitative real-time PCR (qRT–PCR)

RNA from cells was extracted using the Qiagen Mini kit following the manufacturer’s protocol. One microgram of RNA was reverse-transcribed to cDNA by using the Qiagen QuantiTect Reverse Transcription kit, according to the manufacturer’s protocol. Afterwards, the cDNA was diluted 10 times before further use. qRT–PCR was performed using commercially available primers for the studied genes (Table 2) and TaqMan Fast Universal PCR Master Mix (Applied Biosystems). In a total volume of 10, 2 μl of the diluted cDNA was added to 500 nM primers and Fast Master Mix and was pipetted in a 96-well MicroAmp plate (Applied Biosystems). This plate was analyzed on the 7500 Fast Real-time PCR system (Applied Biosystems). Gene transcription was presented as the number of mRNA copies of the gene of interest with respect to the β-actin copies in each sample.

**Table 2:** Primers used for qRT–PCR

Primers	Reference
*PHD1*	Applied Biosystems: Hs01091275
*PHD2*	Applied Biosystems: Hs00254392
*PHD3*	Applied Biosystems: Hs00222966
*β-actin*	IDT: Hs PT39a22214847
*ATM*	IDT: Hs PT56a2596352
*ATR*	IDT: Hs PT56a27318728
*Chk1*	IDT: Hs PT583518318
*Chk2*	IDT: Hs PT56a24327520
*p38α*	IDT: Hs PT5840355791
*DNA-PK*	IDT: Hs PT56a25320213
*XPB*	IDT: Hs PT5815606497

### Immunocytochemistry

HCT116 cells seeded on coverslips in 12-well format were stained according to the manual of the ApopTag® Fluorescein *In Situ* Apoptosis Detection Kit (Merck Millipore), and the apoptotic area was quantified by microscopic analysis with an Olympus BX41 microscope and CellSense imaging software. ApopTag analysis showing the average ± s.e.m. from three biological replicates wherefrom each time three fields were analyzed.

### Cell cycle analysis

Supernatants derived from HCT116 cells were collected, and adherent cells were subsequently trypsinized, added to the supernatant previously collected, and centrifuged at 300 *g* for 5 min. After one wash with PBS, cells were fixed with 1 ml of 70% ethanol. Cells were incubated 2 h or overnight at 4°C, prior to another centrifugation at 300 *g* for 5 min. Supernatant was removed and the pellet was resuspended in 200 μl of PBS, containing 500 μg of RNase (10 mg/ml). About 200 μl of propidium iodide (0.1 mg/ml) was added to a final volume of 400 μl. Samples were incubated for 1–2 h at 37°C and subsequently analyzed by fluorescence-activated cell sorting by using FACS Canto II (BD Bioscience). FACS analysis was performed on three independent experiments wherefrom a representative experiment is shown.

### ELISA nucleosome detection

The nucleosome detection was performed by using the Roche Cell Death Detection ELISA^PLUS^ according to the manufacturer’s protocol. ELISA was performed in two independent experiments with three biological replicates each, wherefrom one representative experiment is shown.

### Stable cell line generation

Full-length Flag-tagged wild-type and different proline mutant forms of p53 were generated by Genscript in a lentiviral vector where p53 is under the RSV promoter as described before (Kaeser *et al*, 2004). A small amount of lentivirus was used to transduce the *p53*^−/−^ HCT116 cells in order to obtain one copy of the lentiviral vector/cell. Selection with puromycin (Sigma-Aldrich) was then performed to achieve a homogeneous population.

### *In vitro* kinase assay

The Flag-tagged p53 proteins were immunoprecipitated from whole cell extracts dissolved in lysis buffer [50 mM Tris–HCl, pH 7.5, 150 mM NaCl, 1% Triton X-100, 2 mM EDTA, 2 mM EGTA, 10 mM Na_4_P_2_O_7_, 1 mM PMSF, and complete protease inhibitor cocktail tablet (Roche)]. To recover Flag p53 immunoprecipitates, 500 μg of total protein was incubated with 2 μg Flag antibody for 1 h at 4°C before Sepharose beads (30 μl per reaction mixture) were added for 12 h. After washing with lysis buffer, the immunocomplexes were washed twice with kinase assay buffer (25 mM MOPS pH 7.2, 25 mM MgCl_2_, 5 mM EGTA, 2 mM EDTA, 250 μM DTT, 6 mM β-glycerophosphate). Then, the samples were incubated in 10 μl kinase assay buffer containing 20 ng of active, recombinant full-length p38 (SignalChem), and 250 μM ATP at 30°C for 25 min. Thereafter, proteins were denaturated by incubating with 4× SDS sample buffer at 95°C for 10 min. Following separation of the proteins by 10% SDS–PAGE, the gel was blotted onto a nitrocellulose membrane, and proteins were analyzed as described before. Experiment was performed twice with the image depicted being a representative of both independent experiments.

### Colony formation assay

*p53*^wt/wt^ and *p53*^−/−^ HCT116 transduced with a doxycycline-inducible shScr or sh*PHD1* construct were treated for 24 h with 1 μg/ml doxycycline (Sigma-Aldrich). Following treatment for 8 h with 300 μM 5-FU or control, the cells were detached and seeded at a cell density of 2,000 cells/6 wells in culture medium containing 1 μg/ml doxycycline. Afterward, colony formation capacity was followed over time and medium was changed every 2–3 days to maintain doxycycline treatment. Colonies were stained with crystal violet and analyzed with ImageJ. Experiments are performed three times, and analysis is a representative experiment.

### Tumor experiments

NMRI nude (Harlan) mice, 6–8-week-old males with a weight between 30 and 40 g, were maintained under SPF conditions with free access to water and food pellets in cages with 4–6 mice/cage. Mice were injected subcutaneously in the flank with 3 × 10^6^ cells of the stably transduced *p53*^wt/wt^ HCT116 *p53*^−/−^ HCT116 shScr or sh*PHD1* cell lines. Tumors were measured in a blinded manner with a caliper, and tumor volume was calculated using the formula: *V* = π × [*d*^2^ × *D*]/6, where *d* is the minor tumor axis and *D* is the major tumor axis. When tumors reached a volume of 200 mm^3^, mice were given access to drinking water containing 1 mg/ml doxycycline in H_2_O containing 5% sucrose until the end of the experiment. Two days later, when tumors reached approximately 250 mm^3^, the mice were randomly assigned to receive either treatment with 100 mg/kg (i.p.) or a control solution was started. Afterwards, mice were treated twice more at days 7 and 14 after treatment start. Eighteen hours after the last dose of 5-FU, mice were euthanized. Housing and all experimental animal procedures were approved by the Institutional Animal Care and Research Advisory Committee of the KU Leuven (P096-2012) and reported according to the ARRIVE guidelines. The experiments were performed two times, with the experiment shown being a representative experiment.

### Statistics

Data entry and all analyses were performed in a blinded fashion. All statistical analyses were performed using GraphPad Prism software. Statistical significance was calculated by two-tailed unpaired *t*-test on two experimental conditions or two-way ANOVA when repeated measures were compared, with *P* < 0.05 considered statistically significant. Exact *P*-values are indicated in the figure legends, except when *P* < 0.0001 as prism does not provide an exact *P*-value below this point. Data were tested for normality using the D’Agostino–Pearson omnibus test (for *n *>* *8) or the Kolmogorov–Smirnov test (for *n *≤* *8) and variation within each experimental group was assessed. Detection of mathematical outliers was performed using the Grubbs’ test in GraphPad. Sample sizes for all experiments were chosen based on previous experiences, and *n*-numbers given in the figure legends always represent biological replicates. All graphs show mean values ± s.e.m.

## Supporting Information

Expanded View Figures PDF

Review Process File
