# Supplementary material for: PHD1 regulates p53-mediated colorectal cancer chemoresistance
Source: EMBO Mol Med. 2015 Aug 19;7(10):1350–65. doi: 10.15252/emmm.201505492 (PMC4604688; doi:10.15252/emmm.201505492)
Supplement: Supplementary file 1 [file emmm0007-1350-sd1.pdf]

Expanded View Figures

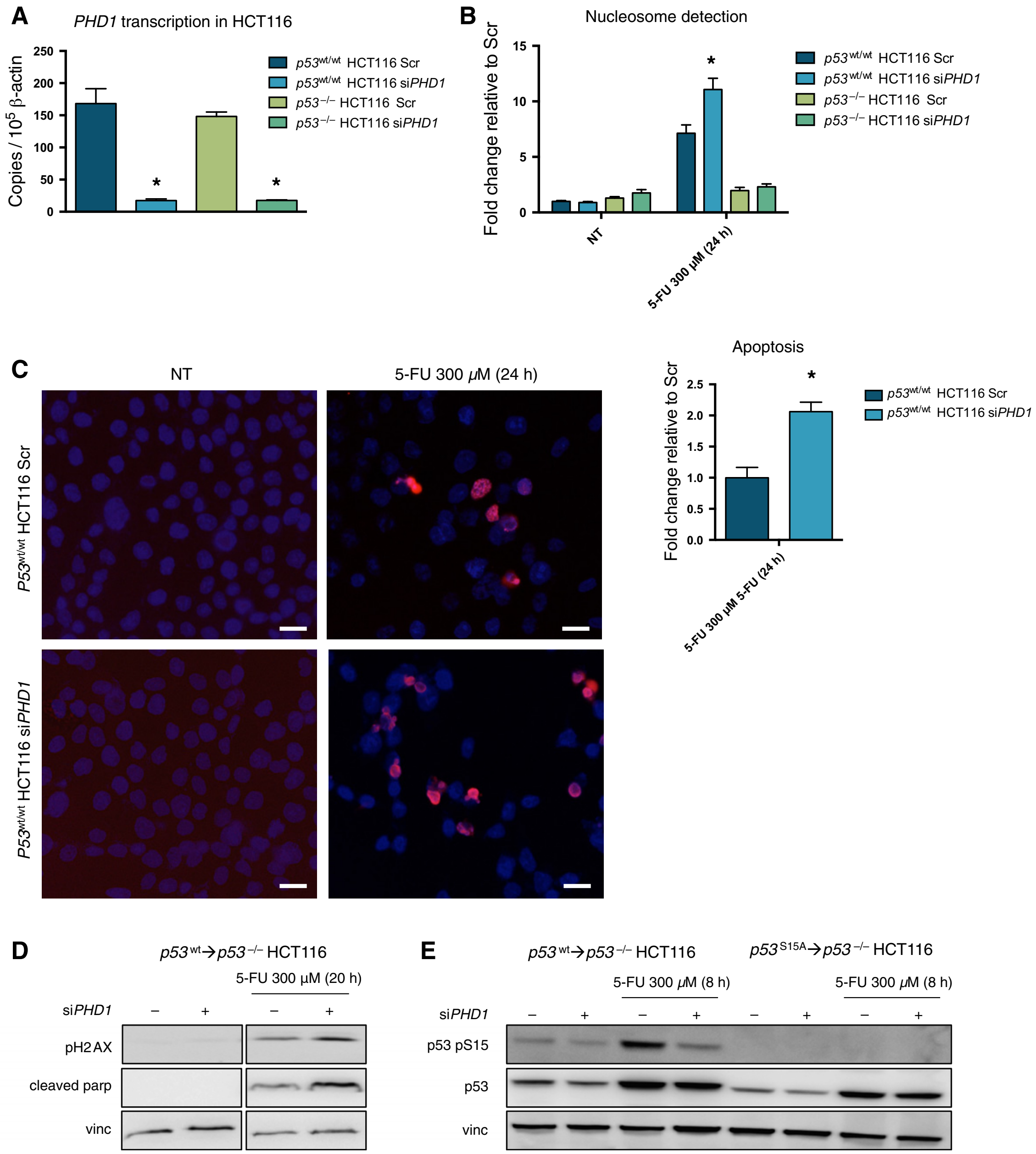

Figure EV1. Silencing of *PHD1* increases cell apoptosis after chemotherapy.

A RNA levels of *PHD1* in $p53^{wt/wt}$ (*P = 0.0027 toward $p53^{wt/wt}$ HCT116 Scr) and $p53^{-/-}$ HCT116 (*P < 0.0001 toward $p53^{-/-}$ HCT116 Scr) silenced for *PHD1*. A two-tailed unpaired t-test was performed with n = 3/group.

B Nucleosome detection as a readout for apoptosis in $p53^{wt/wt}$ and $p53^{-/-}$ HCT116 silenced for *PHD1* after 24 h of 300-μM 5-FU treatment. *P = 0.04 toward the Scr 5-FU-treated condition as tested by a two-tailed unpaired t-test with n = 3/group.

C Apoptosis in HCT116 silenced for *PHD1* and treated with 300 μM 5-FU for 24 h as detected by TUNEL immunocytochemistry and the quantification of these results. Scale bar represents 20 μm. *P = 0.003 toward the Scr 5-FU-treated condition as tested by a two-tailed unpaired t-test with n = 4/group.

D Western blot detection of p53 pS15, p53, and vinculin (vinc) in $p53^{wt}$→$p53^{-/-}$ and $p53^{S15A}$→$p53^{-/-}$ HCT116 cells treated with 300 μM 5-FU for 8 h.

E Parp cleavage, pH2AX, and vinc in $p53^{wt}$→$p53^{-/-}$ HCT116 cells upon 20-h treatment with 300 μM 5-FU.

Data information: Vinc was used as a loading control in (D, E).

© 2015 The Authors

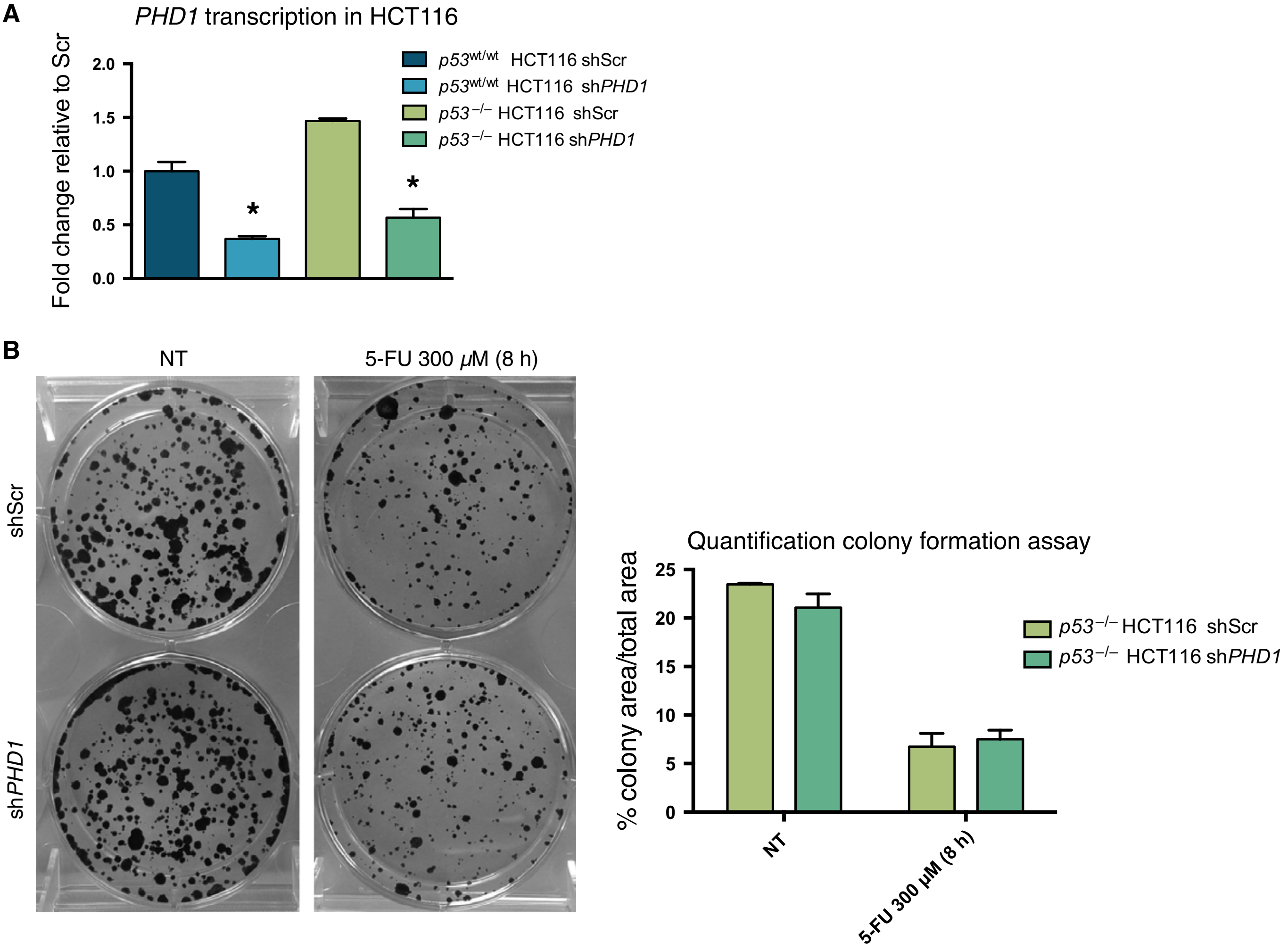

Figure EV2. Colony formation with *p53*$^{-/-}$ HCT116 cells is not altered by *PHD1* silencing.

A Evaluation of *PHD1* transcription by qRT–PCR in *p53*$^{wt/wt}$ (**P* < 0.0001 toward *p53*$^{wt/wt}$ HCT116 shScr) and *p53*$^{-/-}$ HCT116 (**P* < 0.0001 toward *p53*$^{-/-}$ HCT116 shScr) silenced for *PHD1* by stable transduction with a doxycycline-inducible construct and treated with 1 μg/ml doxycycline for 24 h. A two-tailed unpaired *t*-test was performed with n = 6/group.

B Colony formation and quantification in *p53*$^{-/-}$ HCT116 shScr and sh*PHD1* after 8 h of 300 μM 5-FU, showing a similar decrease in colony formation between the two conditions compared to the non-treated cells.

 © 2015 The Authors

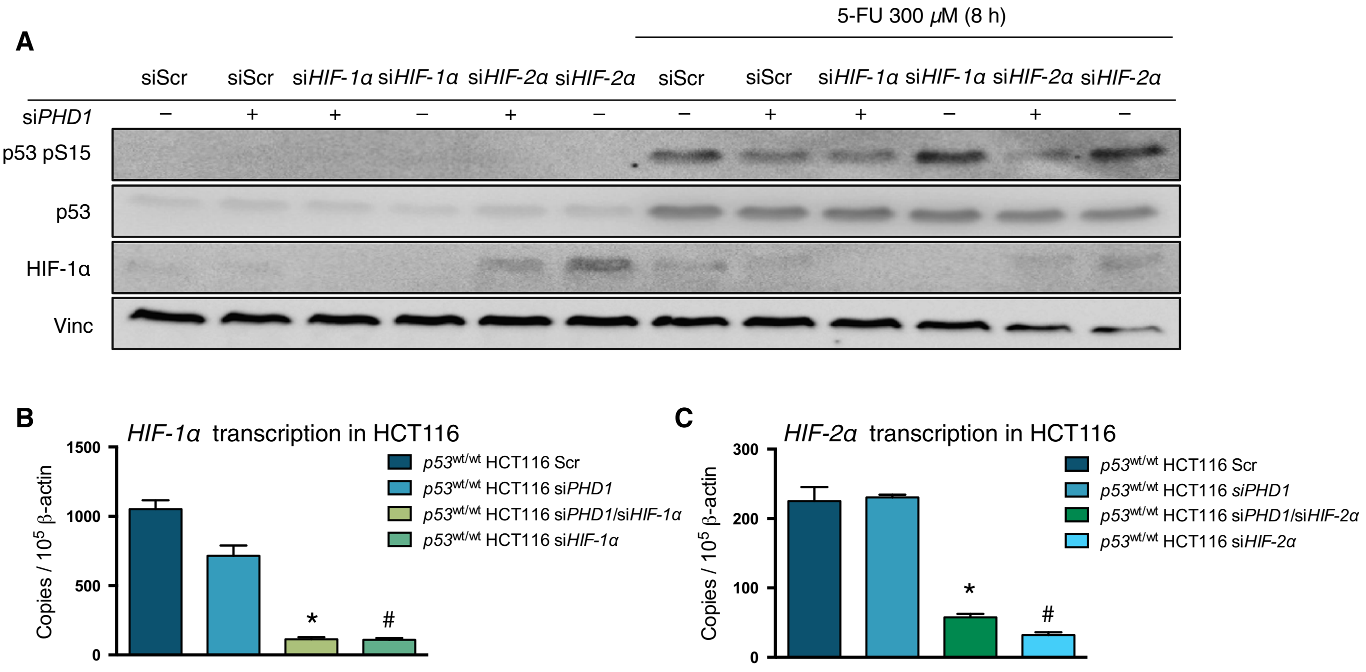

Figure EV3. HIFs do not play a role in the regulation of p53 phosphorylation upon PHD1 silencing.

A Western blot detection of p53 pS15, p53, HIF-1α, and vinculin (vinc) in HCT116 silenced for *PHD1* either alone or in combination with *HIF-1α* or *HIF-2α* silencing.

B, C qRT–PCR for *HIF-1α* (B, *P = 0.0003 and #P = 0.0003 both toward Scr) and *HIF-2α* (C, *P = 0.001 and #P = 0.0007 both toward Scr) in HCT116 silenced for *PHD1* alone or in combination with either of the respective genes. A two-tailed unpaired t-test was performed with n = 3/group.

© 2015 The Authors

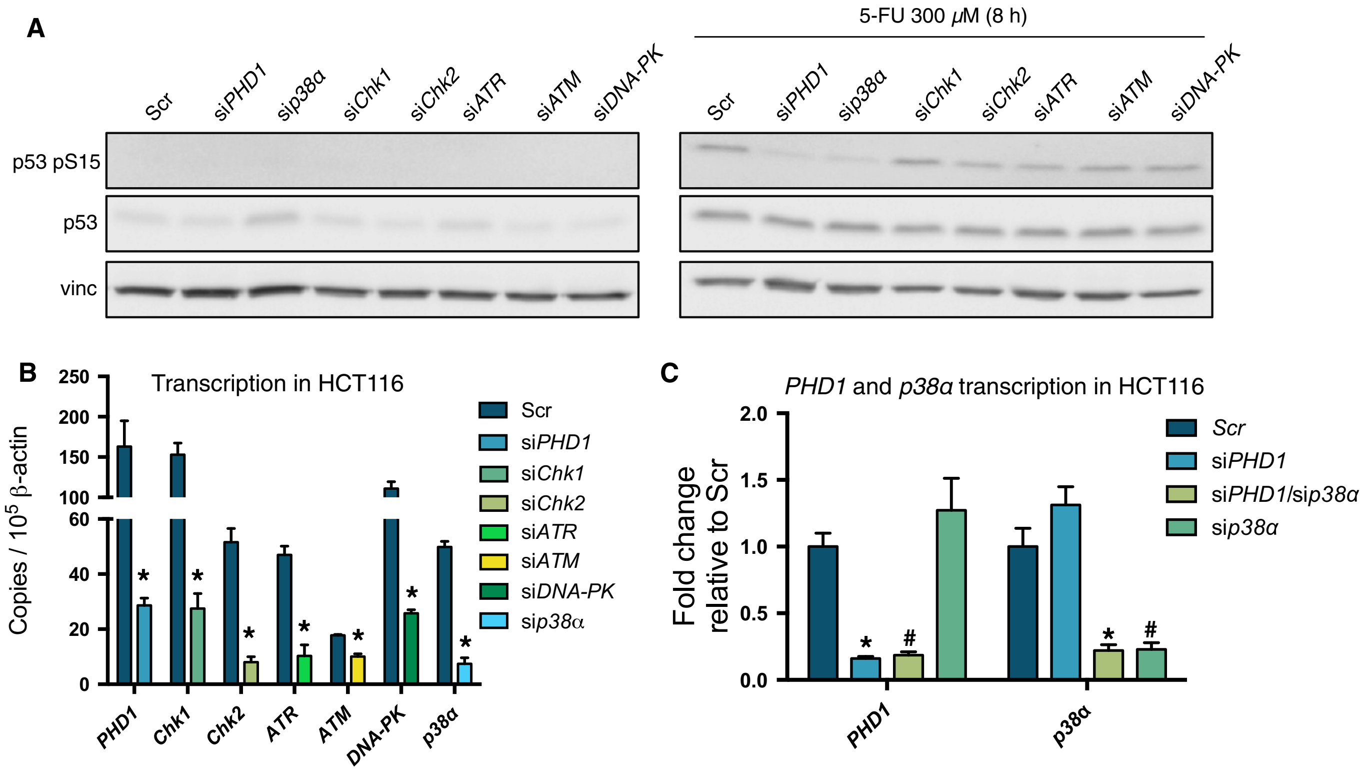

Figure EV4. Silencing efficiency in HCT116 cells.

A Western blot for p53 pS15, p53, and vinculin (vinc) in HCT116 silenced for either *PHD1*, *Chk1*, *Chk2*, *ATR*, *ATM*, *DNA-PK*, and *p38α* and treated with 300 μM 5-FU for 8 h.

B Analysis by qRT–PCR of *PHD1* (*P = 0.01 toward Scr), *Chk1* (*P = 0.007 toward Scr), *Chk2* (*P = 0.002 toward Scr), *ATR* (*P = 0.002 toward Scr), *ATM* (*P = 0.002 toward Scr), *DNA-PK* (*P = 0.001 toward Scr), and *p38α* (*P = 0.0001 toward Scr) transcription in HCT116 upon their respective silencing in comparison with the Scr control. A two-tailed unpaired t-test was performed with n = 3/group.

C Analysis by qRT–PCR of *PHD1* (*$P < 0.0001$ and $^{\#}P$ = 0.0009 both toward Scr by a two-tailed unpaired t-test with n = 6 for Scr and si*PHD1* and n = 3 for siPHD1/sip38α and sip38α) and *p38α* (*P = 0.006 and $^{\#}P$ = 0.02 both toward Scr by a two-tailed unpaired t-test with n = 3/group) upon silencing of these genes in comparison with Scr in HCT116.

 © 2015 The Authors

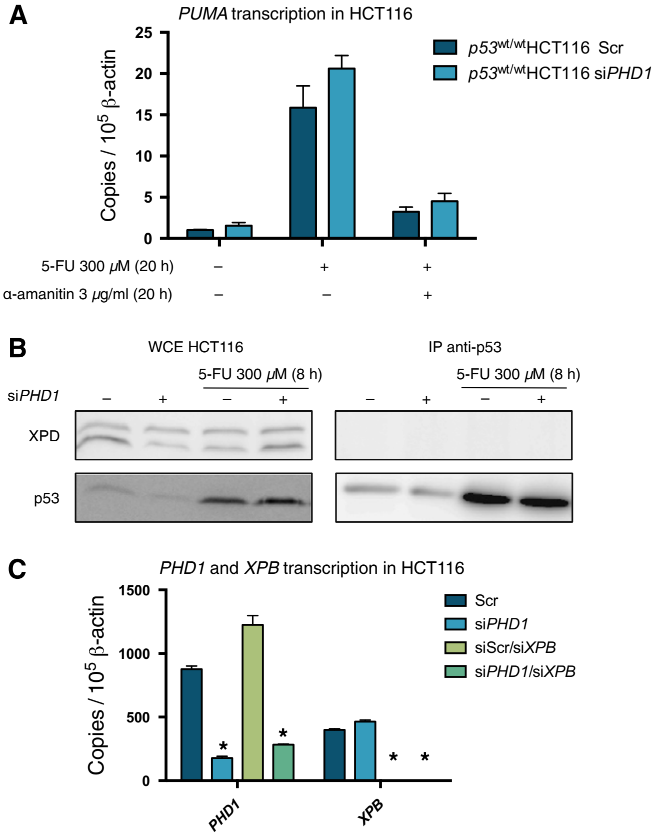

Figure EV5. Transcription-independent regulation of p53 by PHD1.

A Transcriptional analysis of *PUMA* in HCT116 shows that addition of 3 μg/ml α-amanitin can block the induction of *PUMA* after 20 h of 300 μM 5-FU treatment.

B Immunoprecipitation of p53 in *PHD1*-silenced HCT116 which were untreated or treated with 300 μM 5-FU for 8 h, showing no interaction between p53 and XPD.

C Analysis by qRT–PCR of *PHD1* and *XPB* upon silencing of these genes in comparison with Scr in HCT116. *$P < 0.0001$ compared to the Scr control by a two-tailed unpaired t-test with $n = 3$/group.

© 2015 The Authors
