# Supplementary material for: PHD1 regulates p53-mediated colorectal cancer chemoresistance
Source: EMBO Mol Med. 2015 Aug 19;7(10):1350–65. doi: 10.15252/emmm.201505492 (PMC4604688; doi:10.15252/emmm.201505492)
Supplement: Supplementary file 2 [file emmm0007-1350-sd2.pdf]

EMBO Molecular Medicine

PHD1 regulation of p53 causes colorectal cancer resistance to chemotherapy

Sofie Deschoemaeker, Giusy Di Conza, Sergio Lilla , Rosa Martín-Pérez, Daniela Mennerich, Lise Boon, Stefanie Hendrikx, Oliver Maddocks, Christian Marx, Praveen Radhakrishnan, Hans Prenen, Martin A. Schneider, Johanna Myllyharju, Thomas Kietzmann, Karen Vousde, Sara Zanivan and Massimiliano Mazzone

Corresponding author: Massimiliano Mazzone, VIB - KU Leuven

Review timeline:

Submission date:	01 June 2015
Editorial Decision:	11 June 2015
Revision received:	01 July 2015
Editorial Decision:	16 July 2015
Accepted:	24 July 2015

Transaction Report:

(Please note that the manuscript was previously reviewed at another journal and the reports were taken into account in the decision making process at EMBO Molecular Medicine. Since the original reviews are not subject to EMBO's transparent review process policy, the reports and author response cannot be published.)

Editor: Roberto Buccione

1st Editorial Decision 11 June 2015

We have now heard from the expert external advisor whom we asked to help us on making a decision on your manuscript.

The advisor was provided with the full manuscript, the previous Reviewers' comments and your point-by-point rebuttal.

I am pleased to say that s/he agrees that the manuscript would make an interesting and worthy contribution and would support publication pending one fundamental technical concern that s/he would be "unhappy to forego": the need to provide evidence for endogenous interaction between PHD1 and p53, As s/he adds, "Otherwise, it remains very possible that the effects are through hydroxylation of some other protein (p38?) that talks to p53, rendering the mechanism presently too vague". The advisor indeed states "This was a weakness already in the original Ms., but it becomes an even more meaningful one now that the authors have removed the claims and information about direct propyl hydroxylation of p53 by PHD1. In their rebuttal, the authors state that the available antibodies are not good enough. I think that they should be requested to try harder... I get the impression that there are quite a number of PHD1 antibodies on the market..."

In conclusion, after internal discussion we agreed that, should you be able to provide this piece of evidence, we would be happy to proceed with your manuscript without further revision other than going back to the advisor for a final check. Needless to say, should you in the meanwhile have obtained further data demonstrating p53 hydroxylation, this would do too.

I look forward to seeing a revised form of your manuscript as soon as possible.

1st Revision - authors' response 01 July 2015

I am pleased to say that s/he agrees that the manuscript would make an interesting and worthy contribution and would support publication pending one fundamental technical concern that s/he would be "unhappy to forego": the need to provide evidence for endogenous interaction between PHD1 and p53, As s/he adds, "Otherwise, it remains very possible that the effects are through hydroxylation of some other protein (p38?) that talks to p53, rendering the mechanism presently too vague". The advisor indeed states "This was a weakness already in the original Ms., but it becomes an even more meaningful one now that the authors have removed the claims and information about direct propyl hydroxylation of p53 by PHD1. In their rebuttal, the authors state that the available antibodies are not good enough. I think that they should be requested to try harder... I get the impression that there are quite a number of PHD1 antibodies on the market..."

In conclusion, after internal discussion we agreed that, should you be able to provide this piece of evidence, we would be happy to proceed with your manuscript without further revision other than going back to the advisor for a final check. Needless to say, should you in the meanwhile have obtained further data demonstrating p53 hydroxylation, this would do too.

I look forward to seeing a revised form of your manuscript as soon as possible.

As you will appreciate, we were able to provide evidence for the endogenous interaction between p53 and PHD1 in the colorectal cancer cell line HCT116 (Fig 4D). In this assay, we included several controls to support our findings, amongst them the silencing of PHD1 and p53, which reduces by 85%, but not entirely the PHD1 levels detected in the pull-down and this is due to the residual PHD1 and p53 present after gene knockdown. The silencing of PHD1 demonstrates that indeed the bands detected are PHD1, whereas the silencing of p53 provides evidence that the PHD1 is not bound in an unspecific way to the beads. This is also further supported by the lack of PHD1 detection after immunoprecipitation of wild type HCT116 lysates with an IgG control antibody. Overall, we hope that this experiment is satisfactory.

2nd Editorial Decision 16 July 2015

Thank you for the submission of your revised manuscript to EMBO Molecular Medicine.

Unfortunately, we were unable to obtain a timely evaluation from the Reviewer who was asked to re-assess you revised manuscript. We thus decided to proceed with an editorial decision. After careful evaluation and further discussion with my colleagues, I am now pleased to inform you that we will be able to accept your manuscript pending the following final amendments:

1) Please specify the gender of the animals used in your experiments.
2) We encourage the publication of source data, particularly for electrophoretic gels and blots, with the aim of making primary data more accessible and transparent to the reader. Would you be willing to provide a PDF file per figure that contains the original, uncropped and unprocessed scans of all or at least the key gels used in the manuscript? The PDF files should be labeled with the appropriate figure/panel number, and should have molecular weight markers; further annotation may be useful but is not essential. The PDF files will be published online with the article as supplementary "Source Data" files. If you have any questions regarding this just contact me.

I have also gone through your text and made some suggested changes (see attached). These mostly concern minor copy-editing issues in the "Abstract" and and "The Paper Explained" sections of your manuscript, and some suggestions for alternative titles. I would appreciate it if you could work from this version when preparing your revision. If you have any problems opening the file or tracking the changes, please let me know. Please remember to include 5 keywords in the Title page.

Please submit your revised manuscript within two weeks at the latest. The sooner you provide the next, final revision, the sooner we will be able to proceed with acceptance.
