# Supplementary figures and images for: PHD1 regulates p53-mediated colorectal cancer chemoresistance
Source: EMBO Mol Med. 2015 Aug 19;7(10):1350–65. doi: 10.15252/emmm.201505492 (PMC4604688; doi:10.15252/emmm.201505492)

Source Data Fig 1

Fig 1A

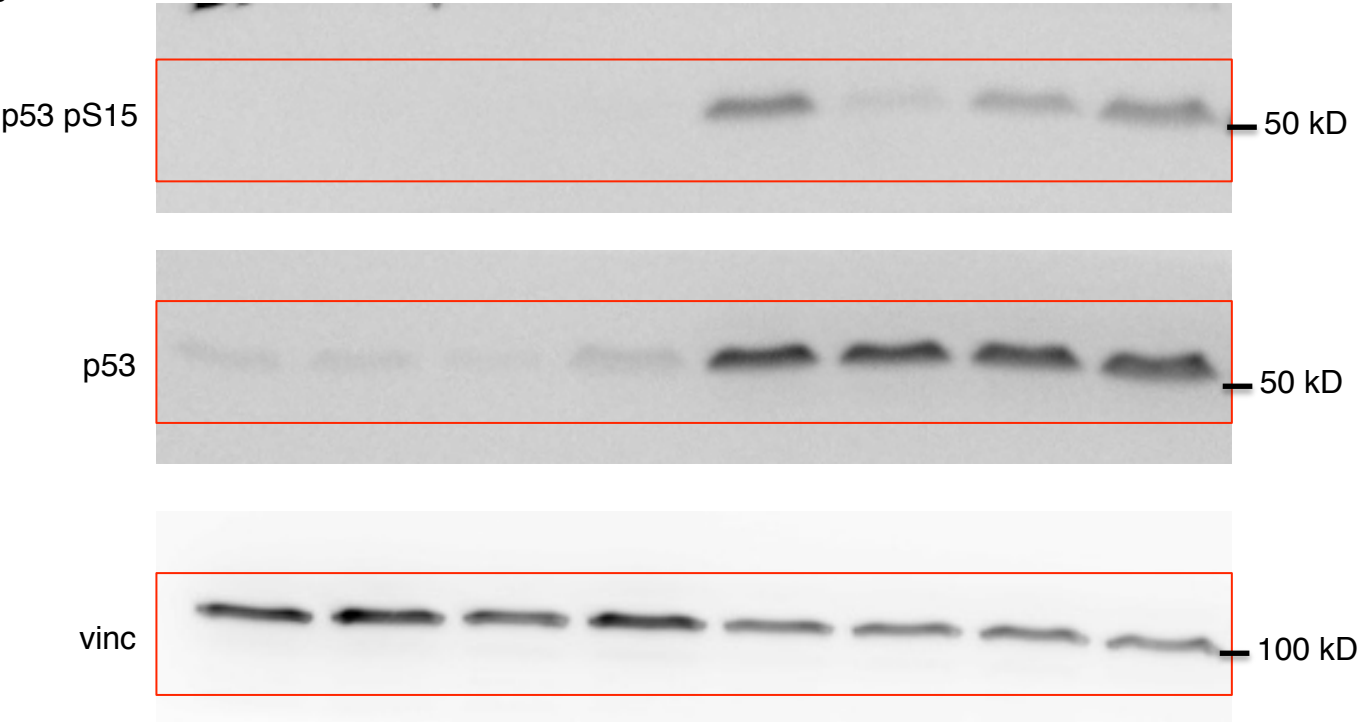

Fig 1C

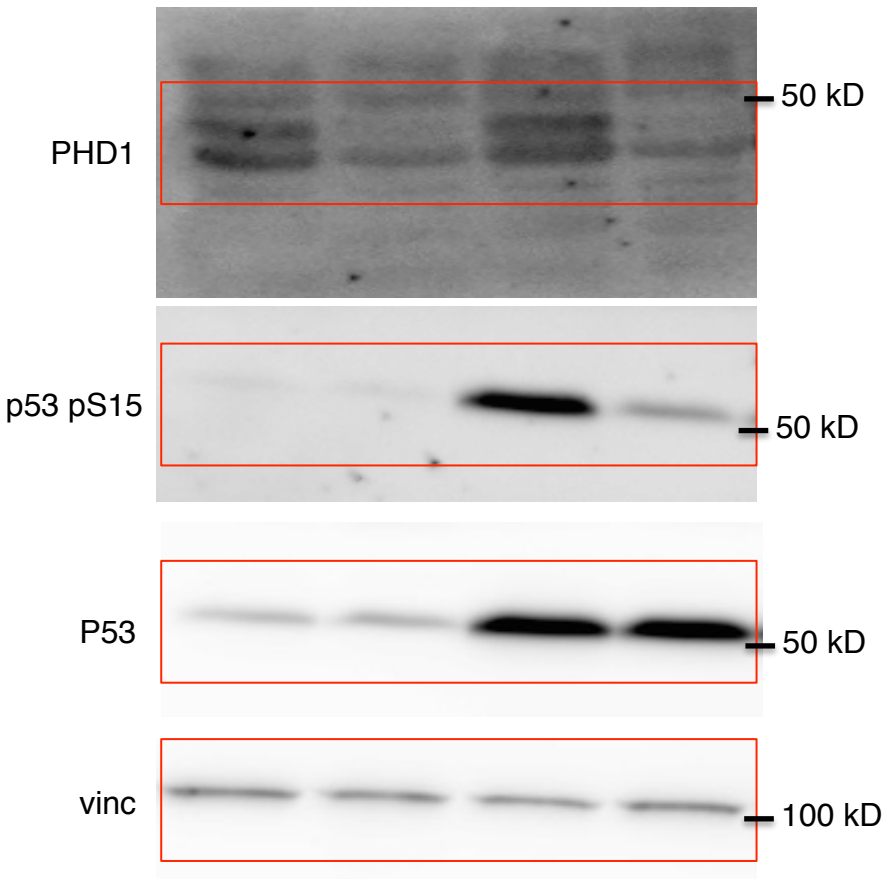

Fig 1E

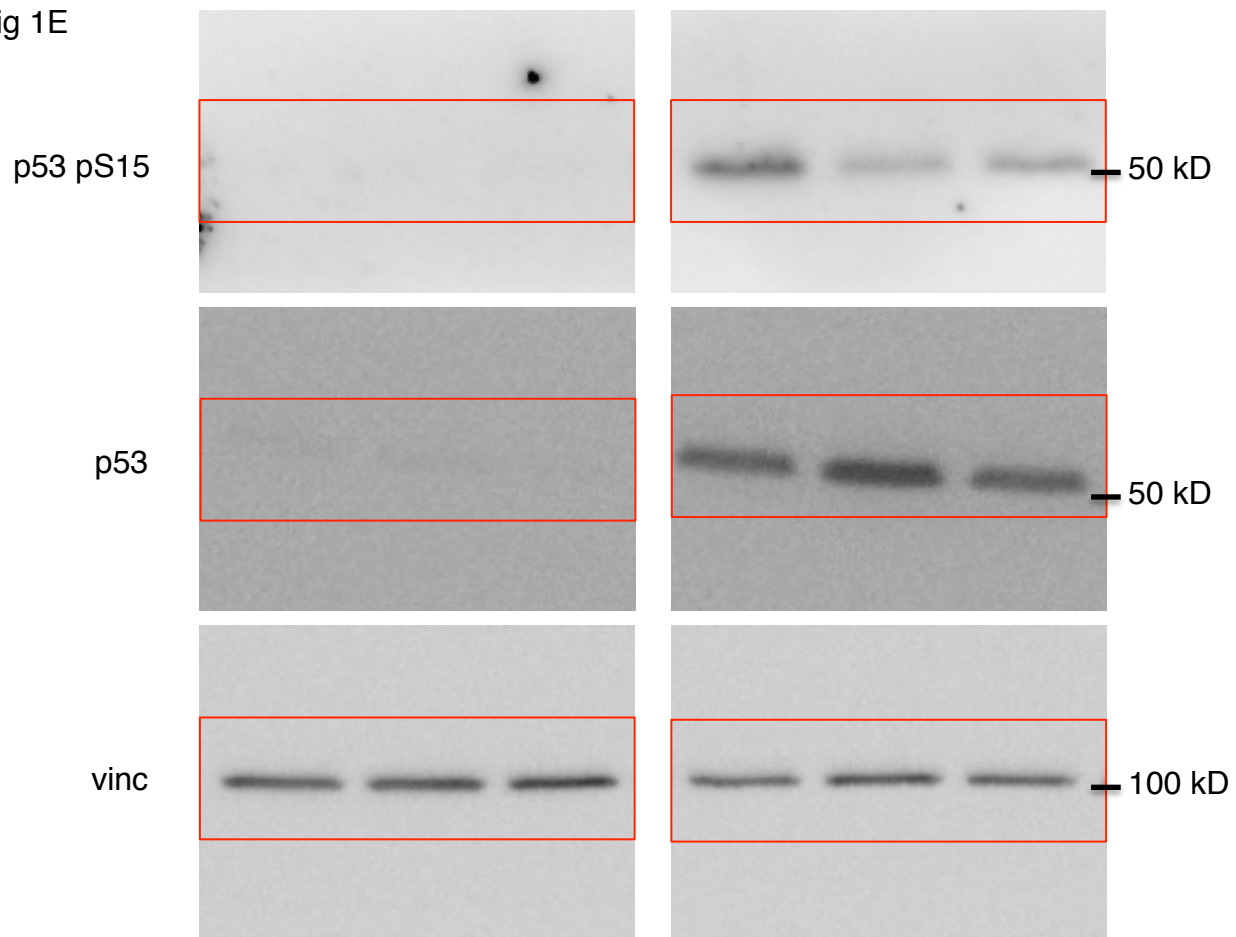

Fig 1F

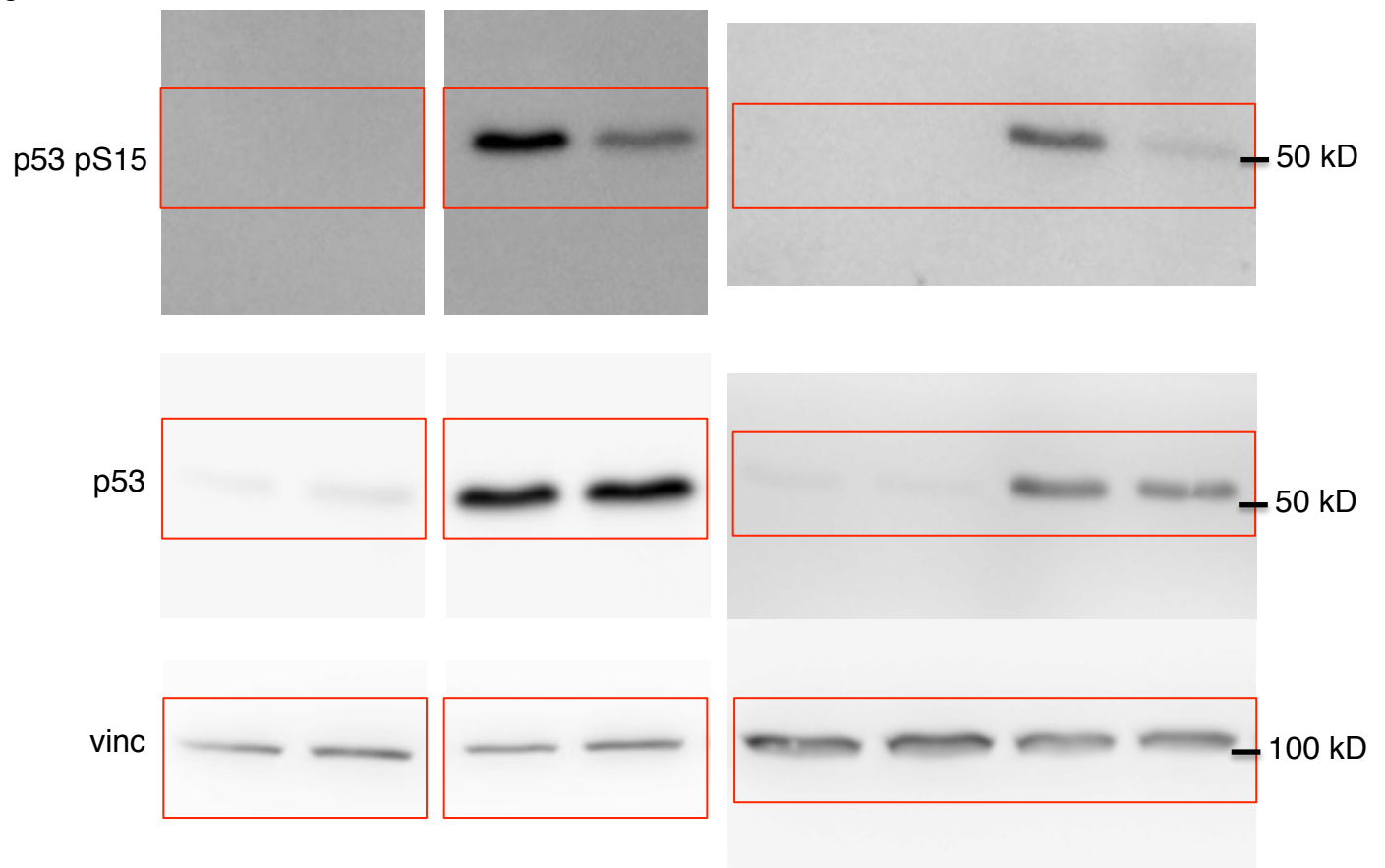

Fig 1H

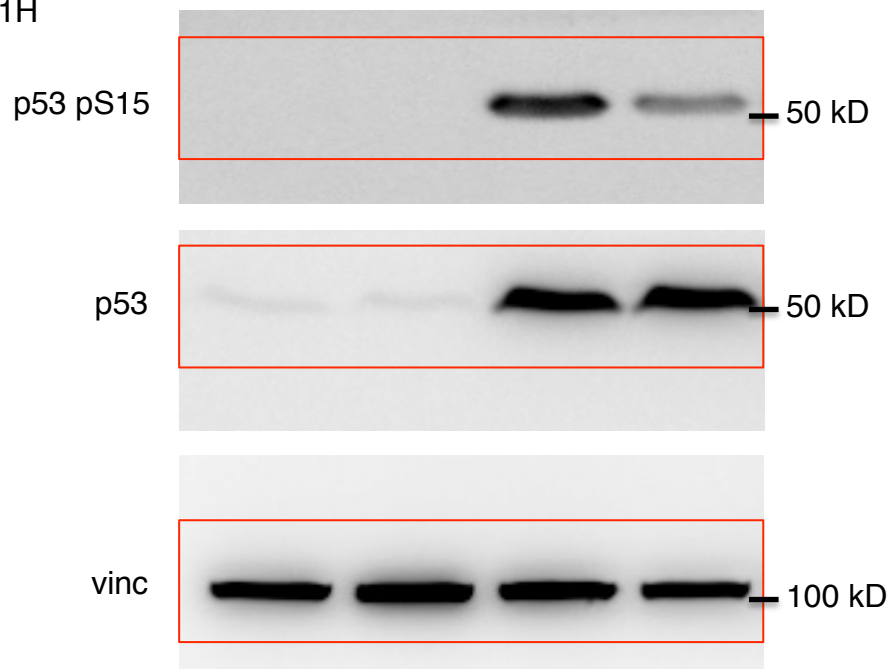

Supplement: Supplementary file 3 [file emmm0007-1350-sd3.pdf]

Source Data Fig 4

Fig 4A

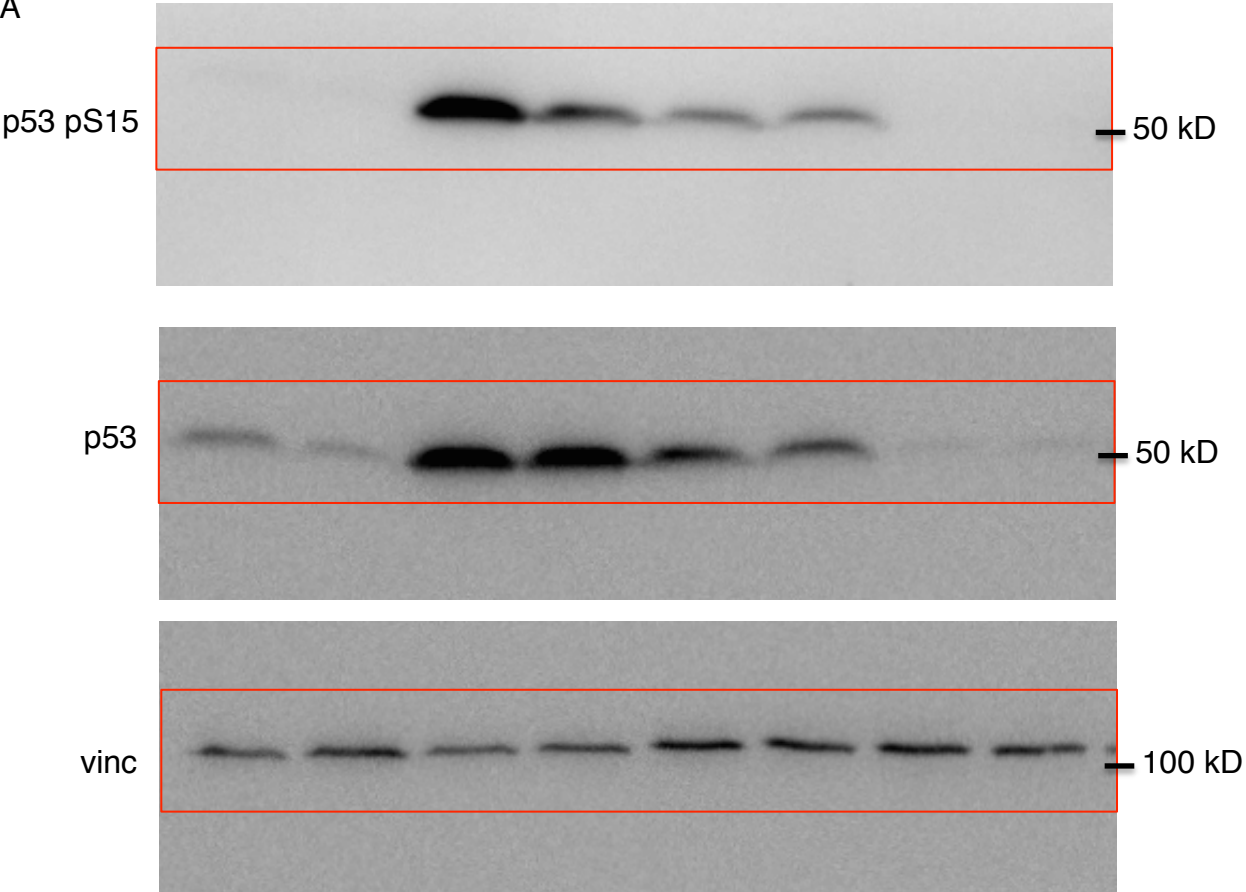

Fig 4B

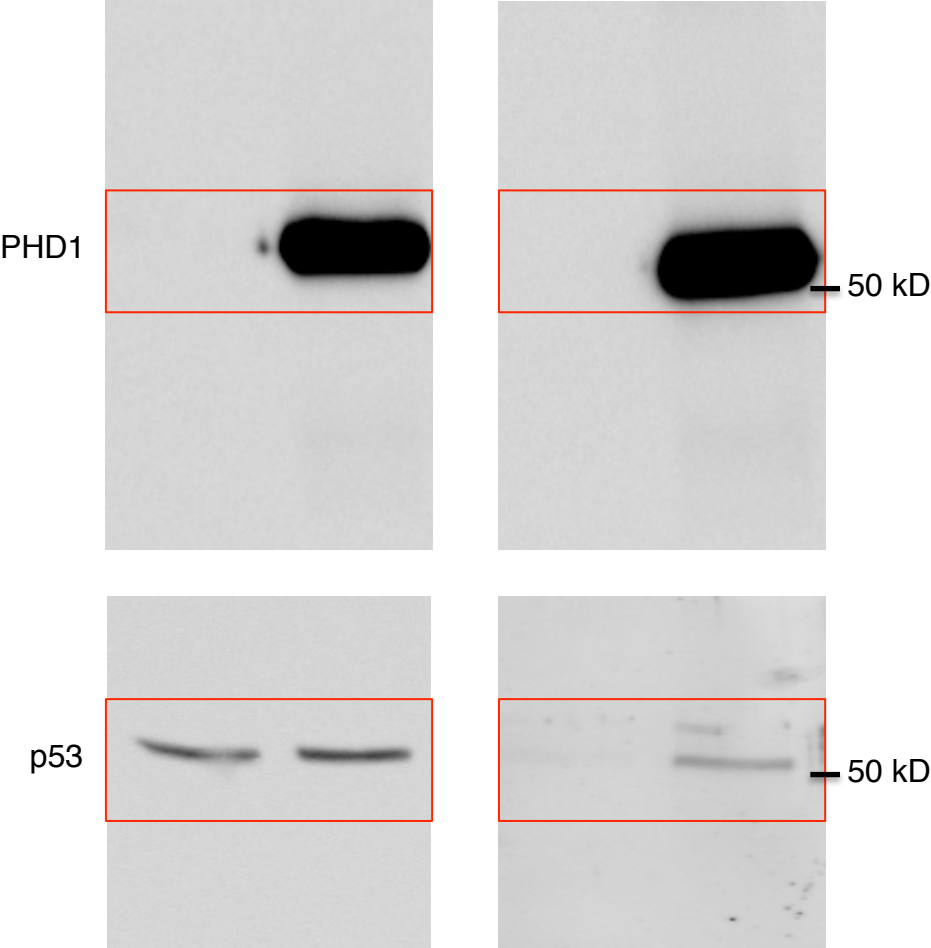

Fig 4C

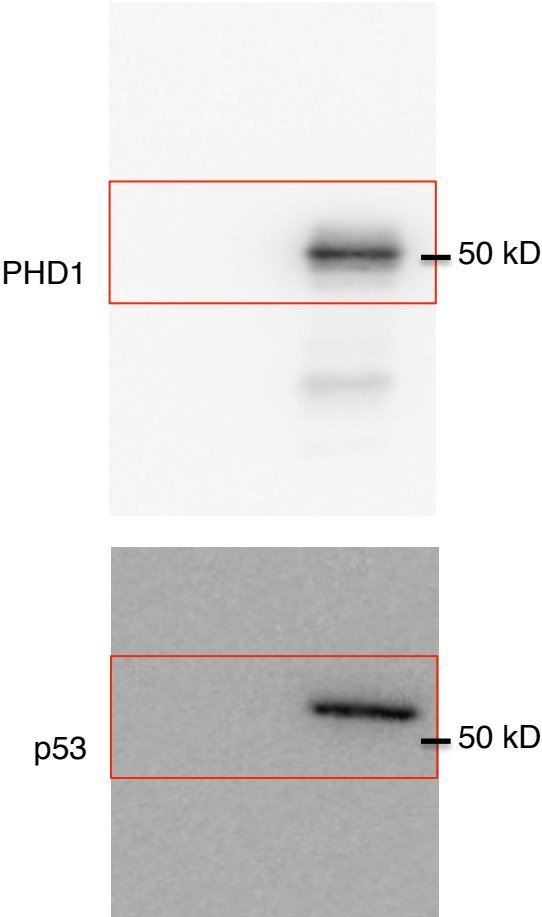

Fig 4D

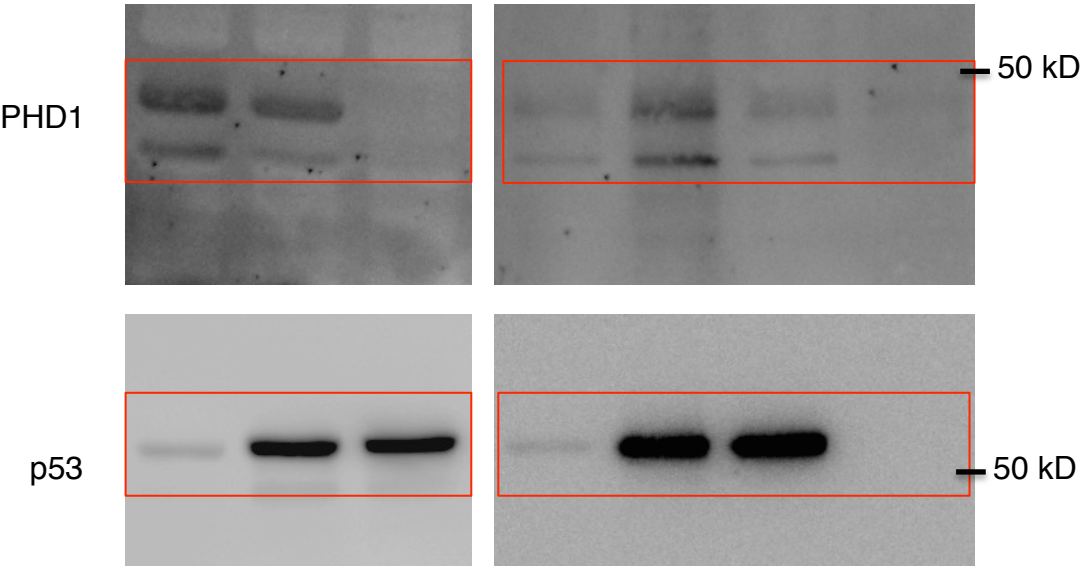

Supplement: Supplementary file 5 [file emmm0007-1350-sd5.pdf]

Source Data Fig 5

Fig 5A

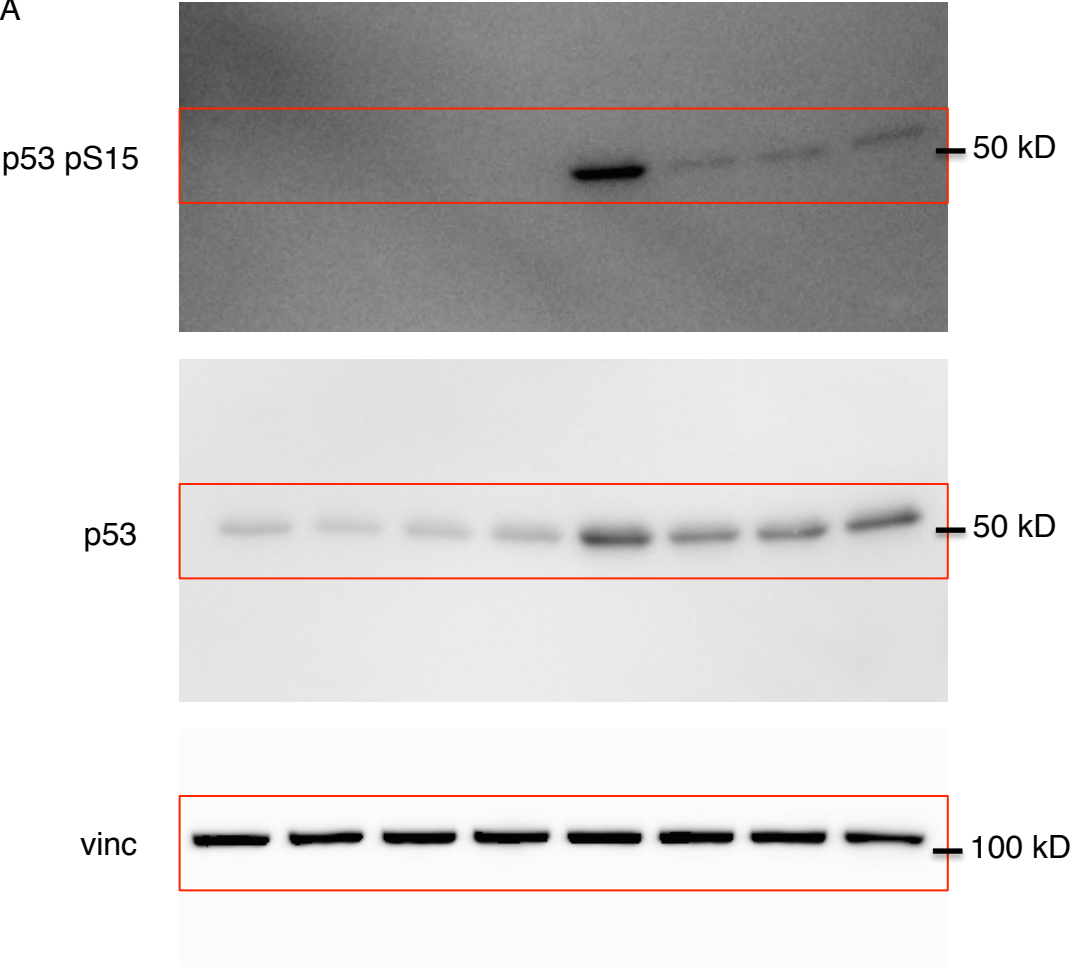

Fig 5B

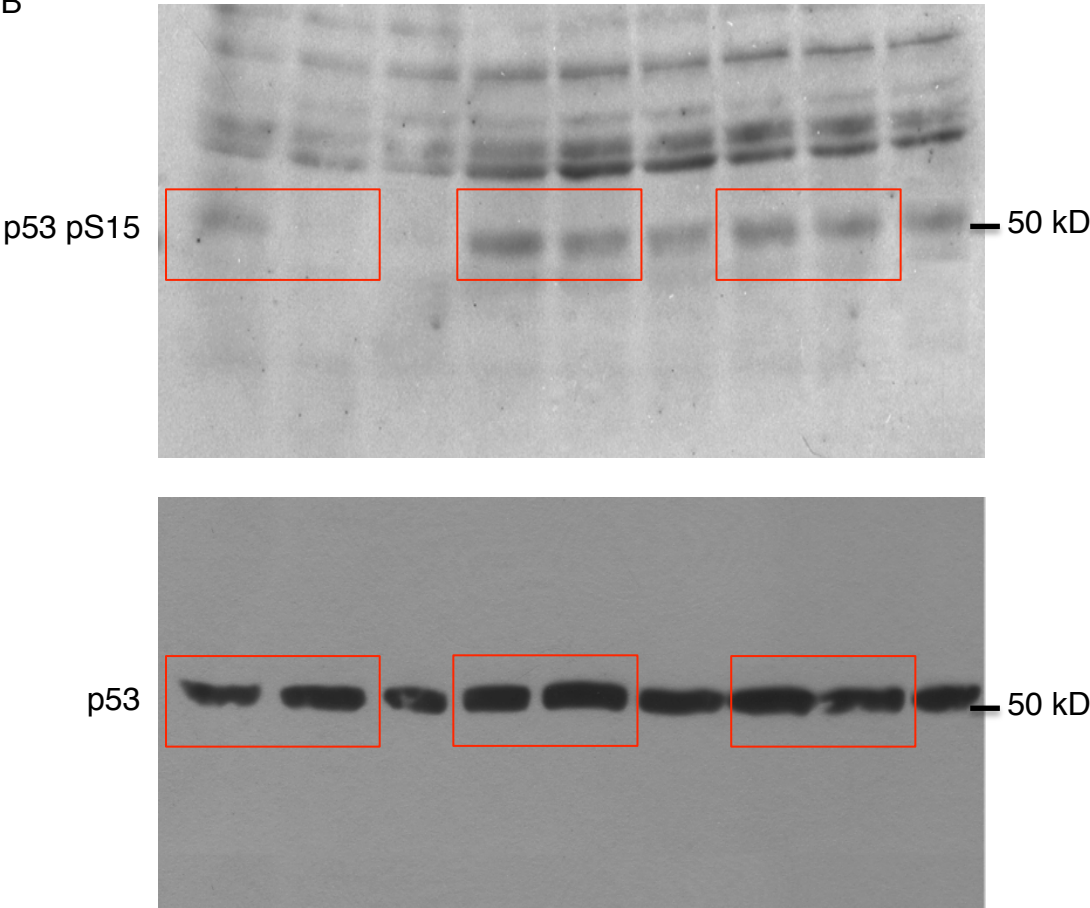

Fig 5C

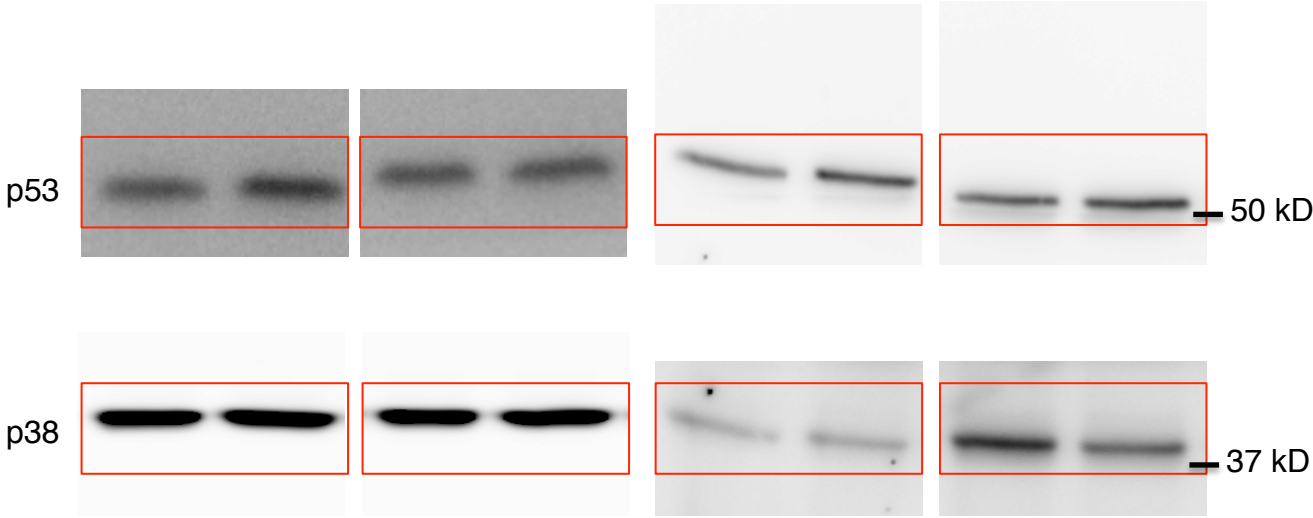

Supplement: Supplementary file 6 [file emmm0007-1350-sd6.pdf]

Source Data Fig 6

Fig 6B

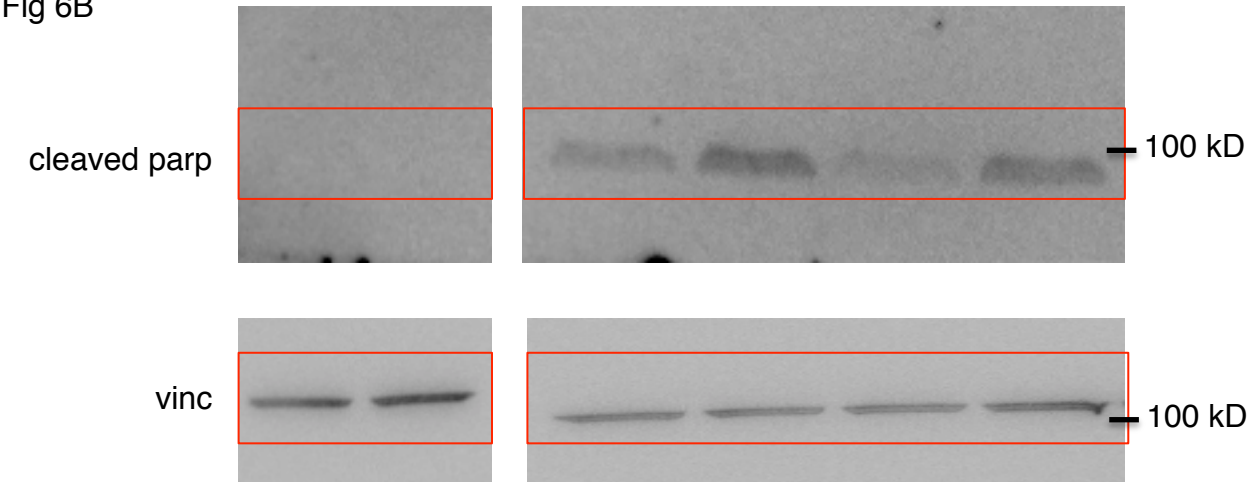

Fig 6D

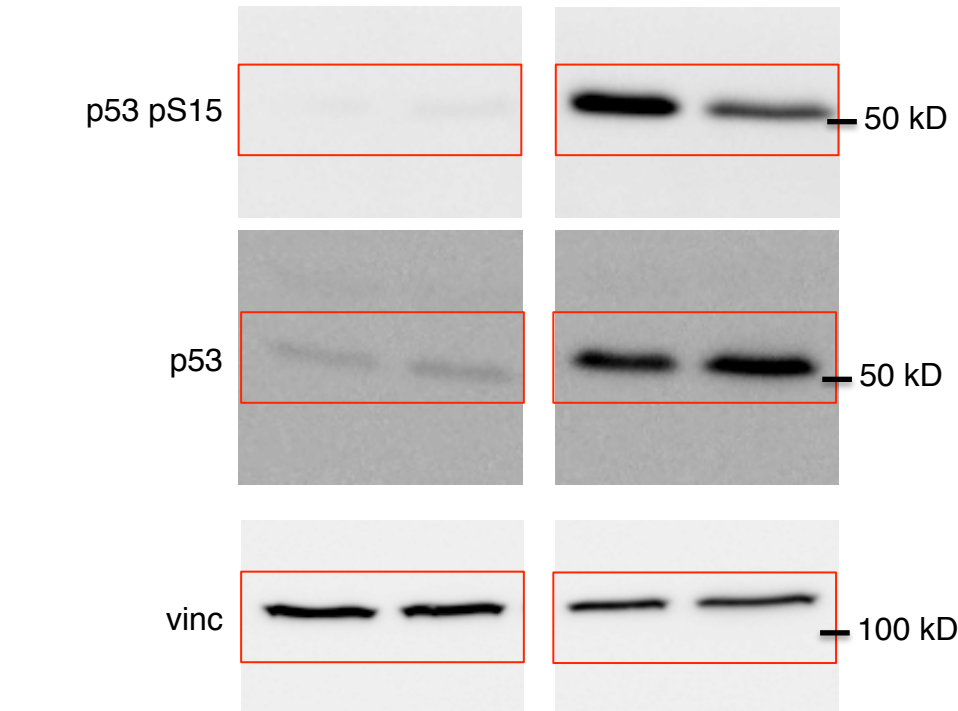

Fig 6E

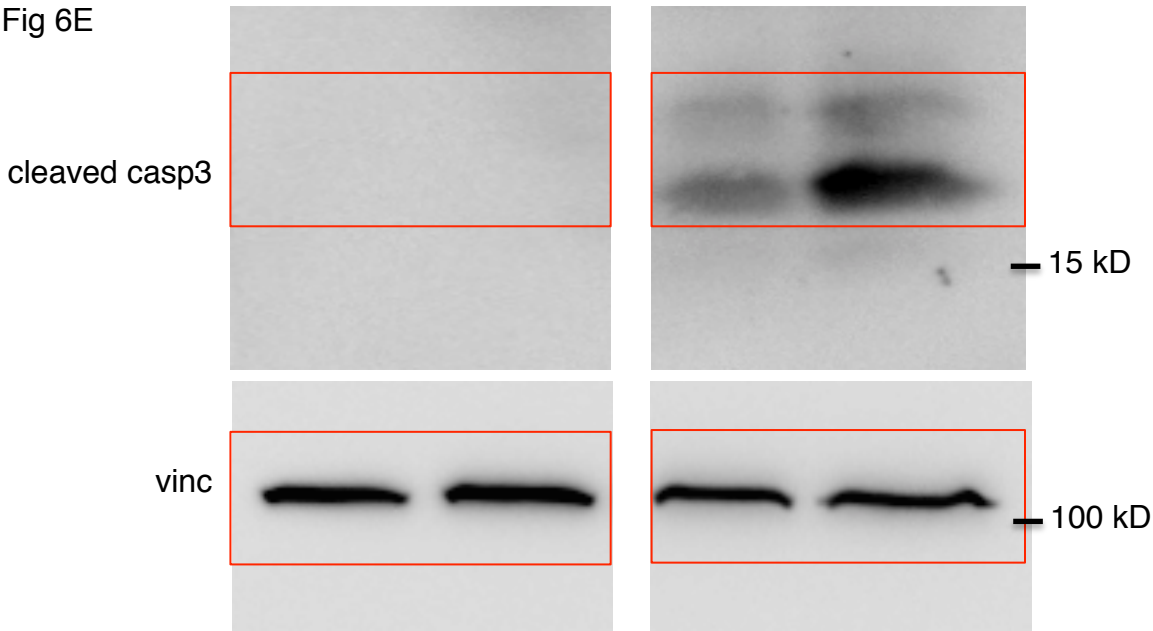

Supplement: Supplementary file 7 [file emmm0007-1350-sd7.pdf]

Source Data Fig 7

Fig 7A

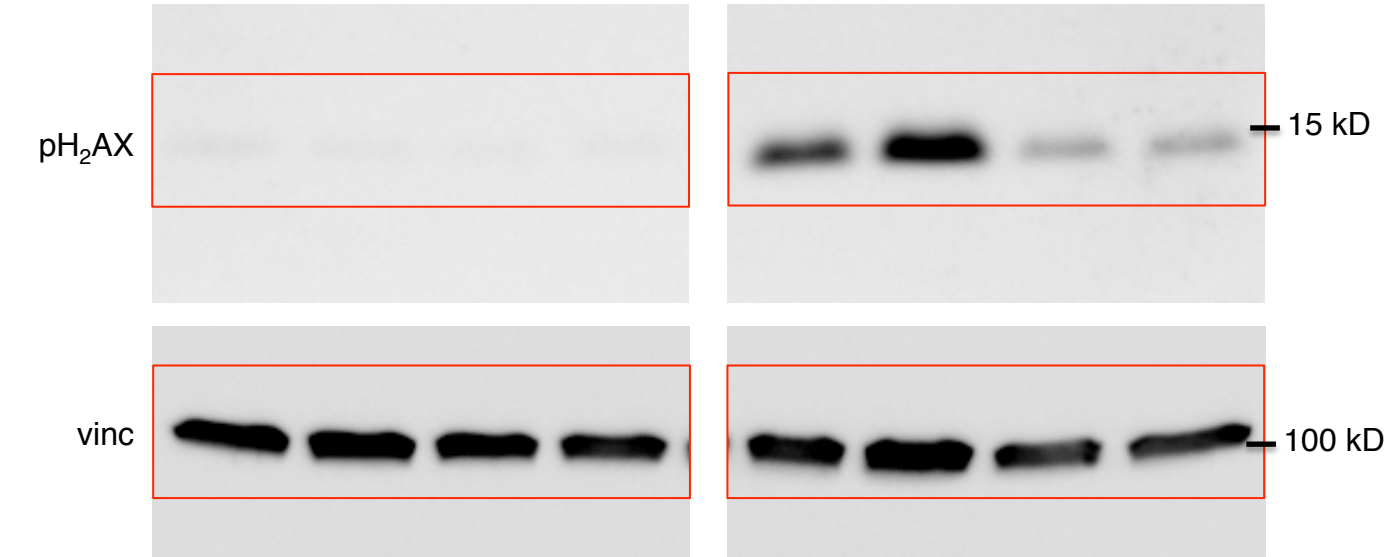

Fig 7B

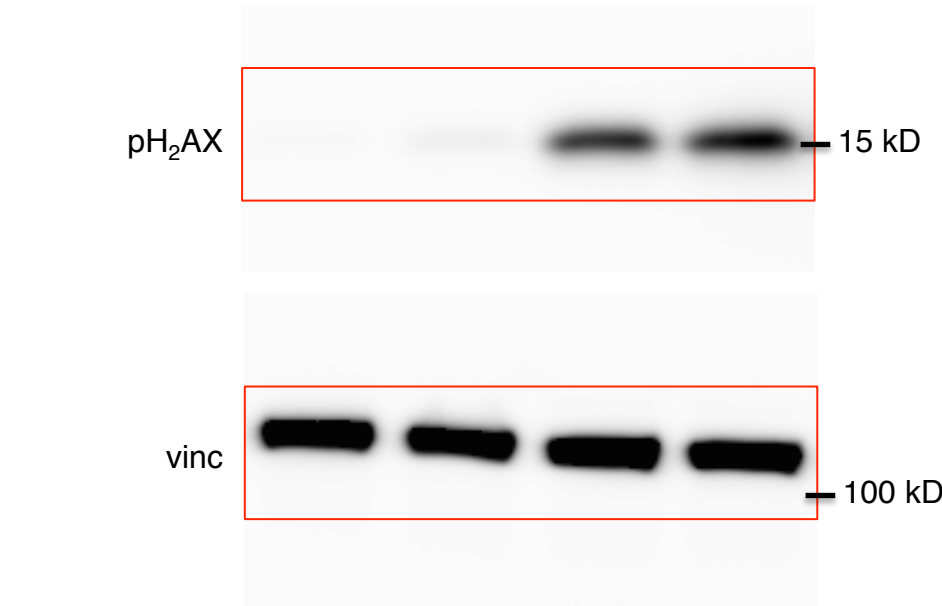

Fig 7C

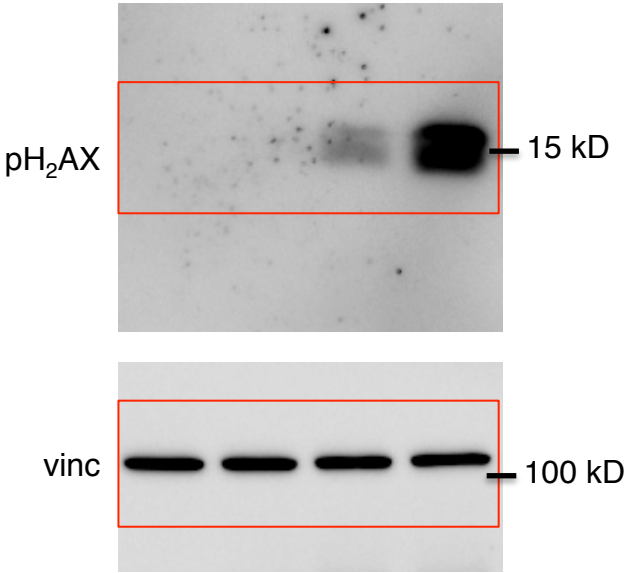

Fig 7D

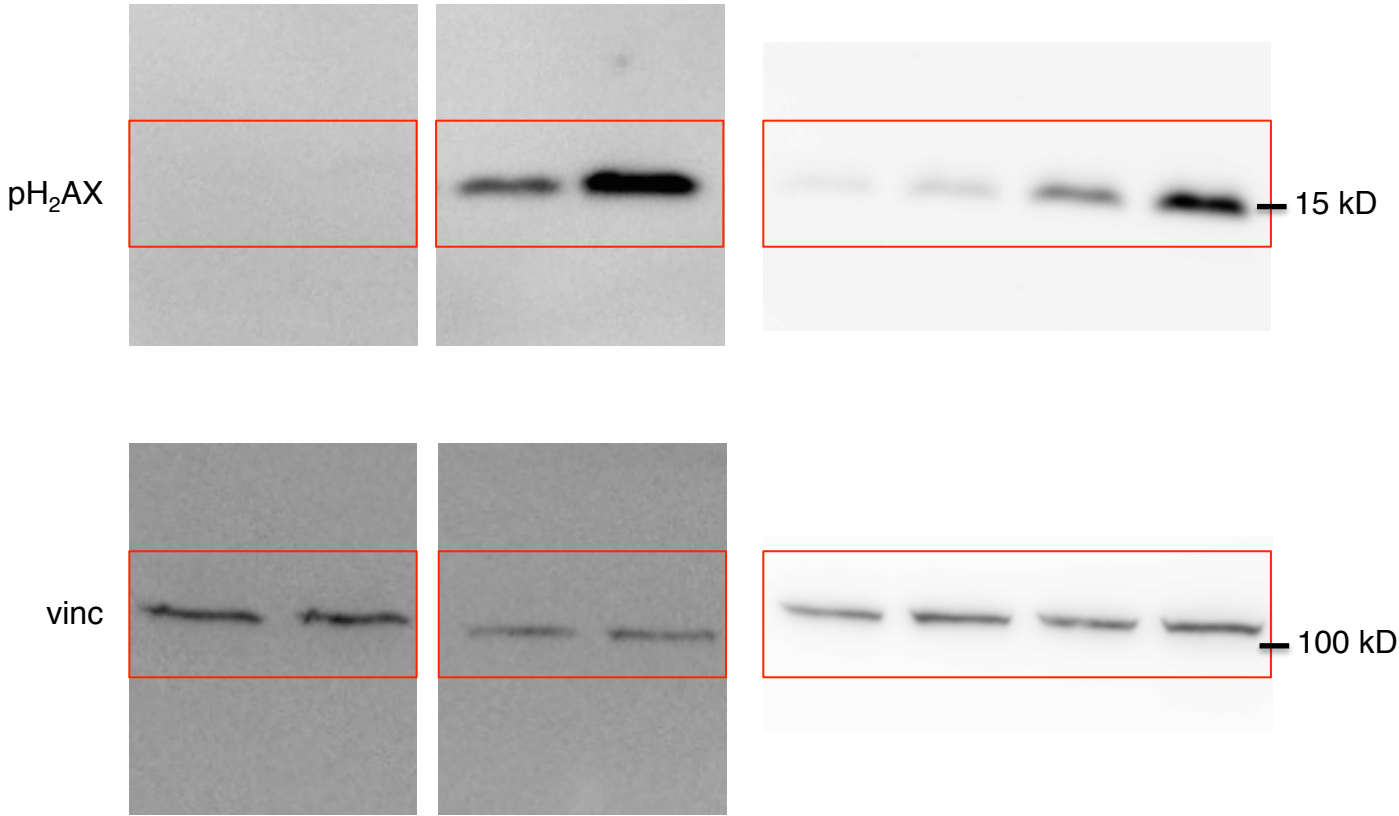

Fig 7E

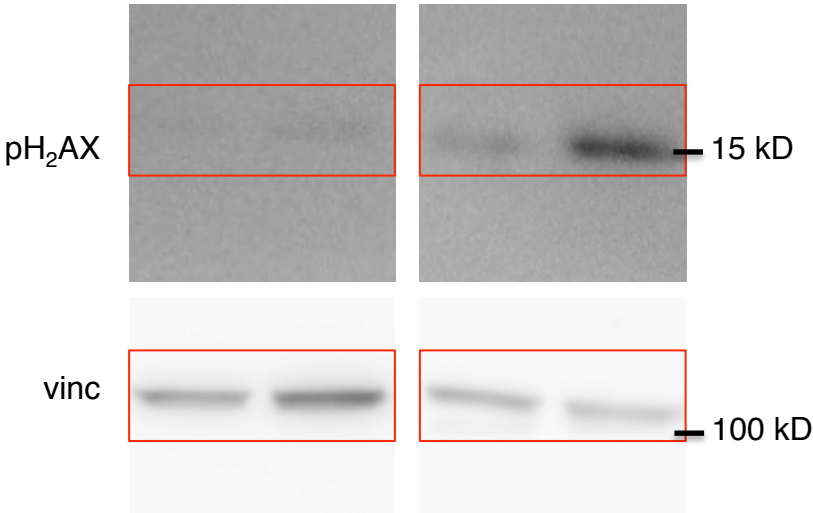

Fig 7F

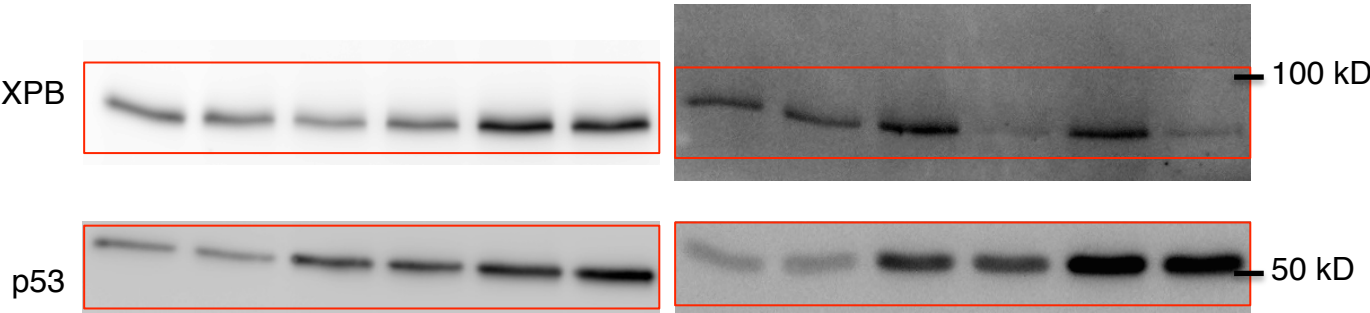

Fig 7G

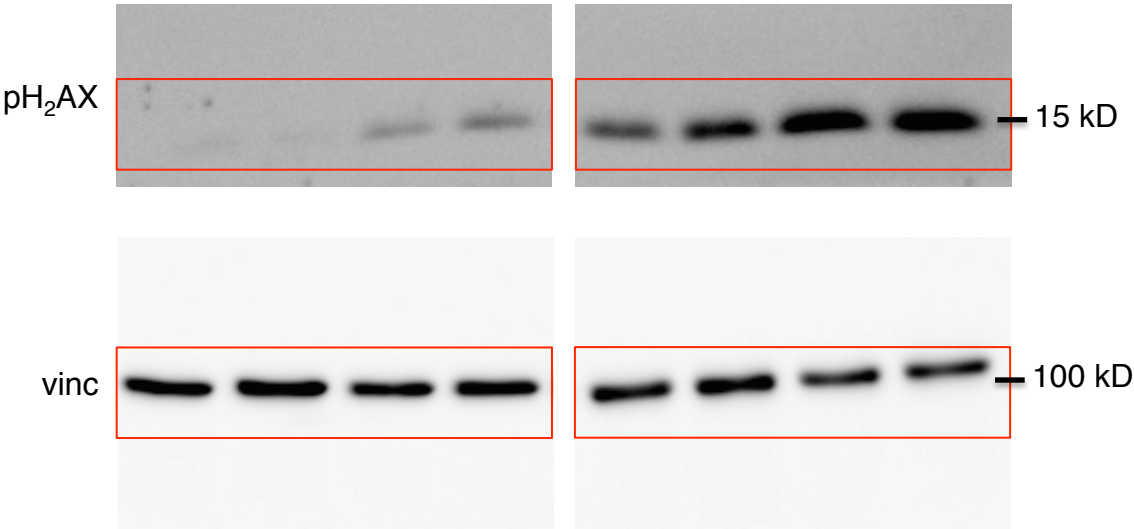

Supplement: Supplementary file 8 [file emmm0007-1350-sd8.pdf]
