# Supplementary material for: PHD1 regulates p53-mediated colorectal cancer chemoresistance
Source: EMBO Mol Med. 2015 Aug 19;7(10):1350–65. doi: 10.15252/emmm.201505492 (PMC4604688; doi:10.15252/emmm.201505492)
Supplement: Supplementary file 4 [file emmm0007-1350-sd4.pdf]

Source Data Fig 2

Fig 2A

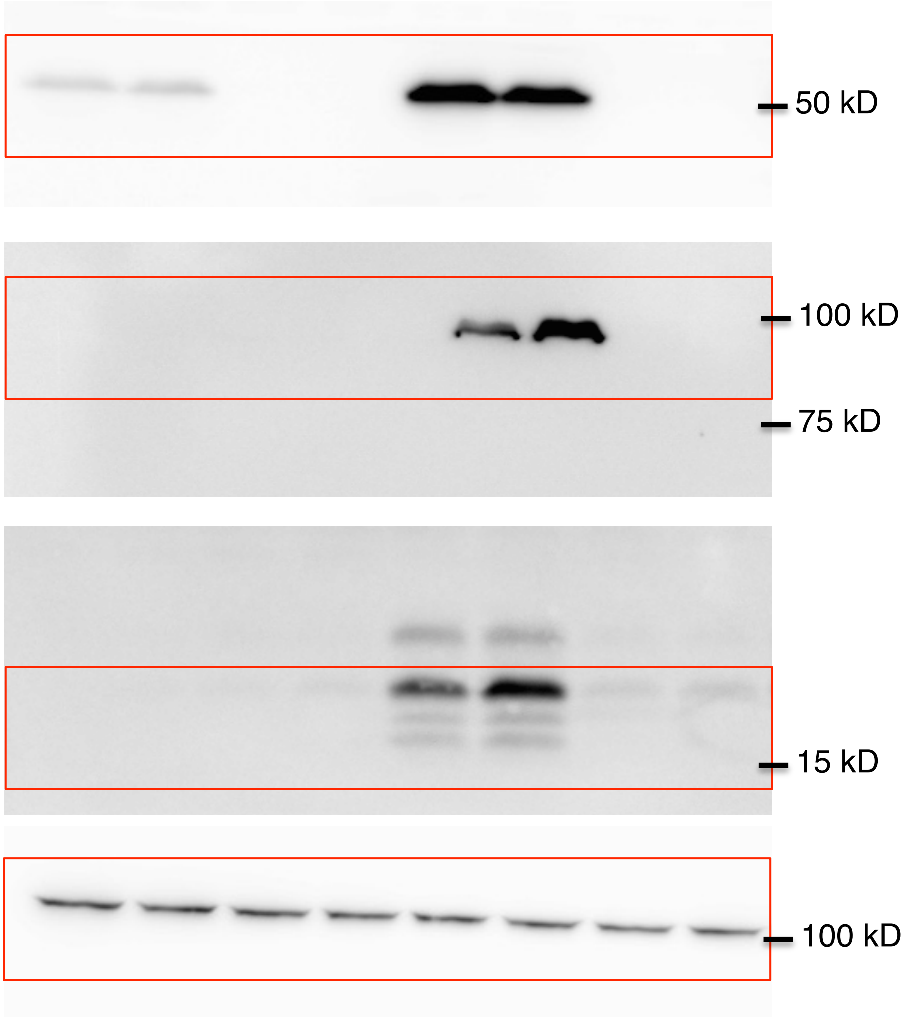

Fig 2C

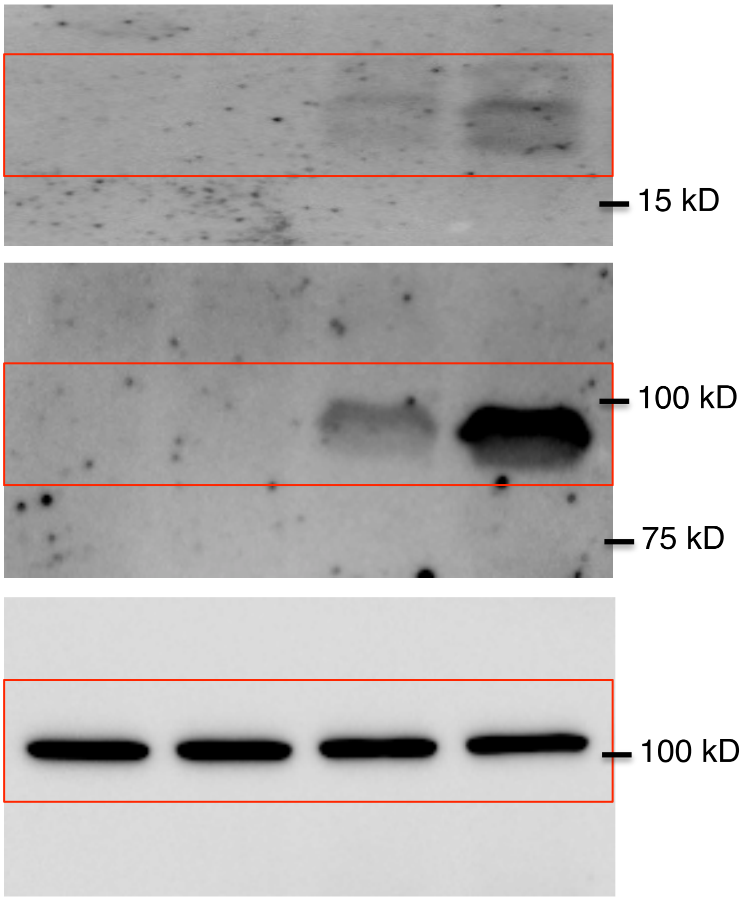

Fig 2D

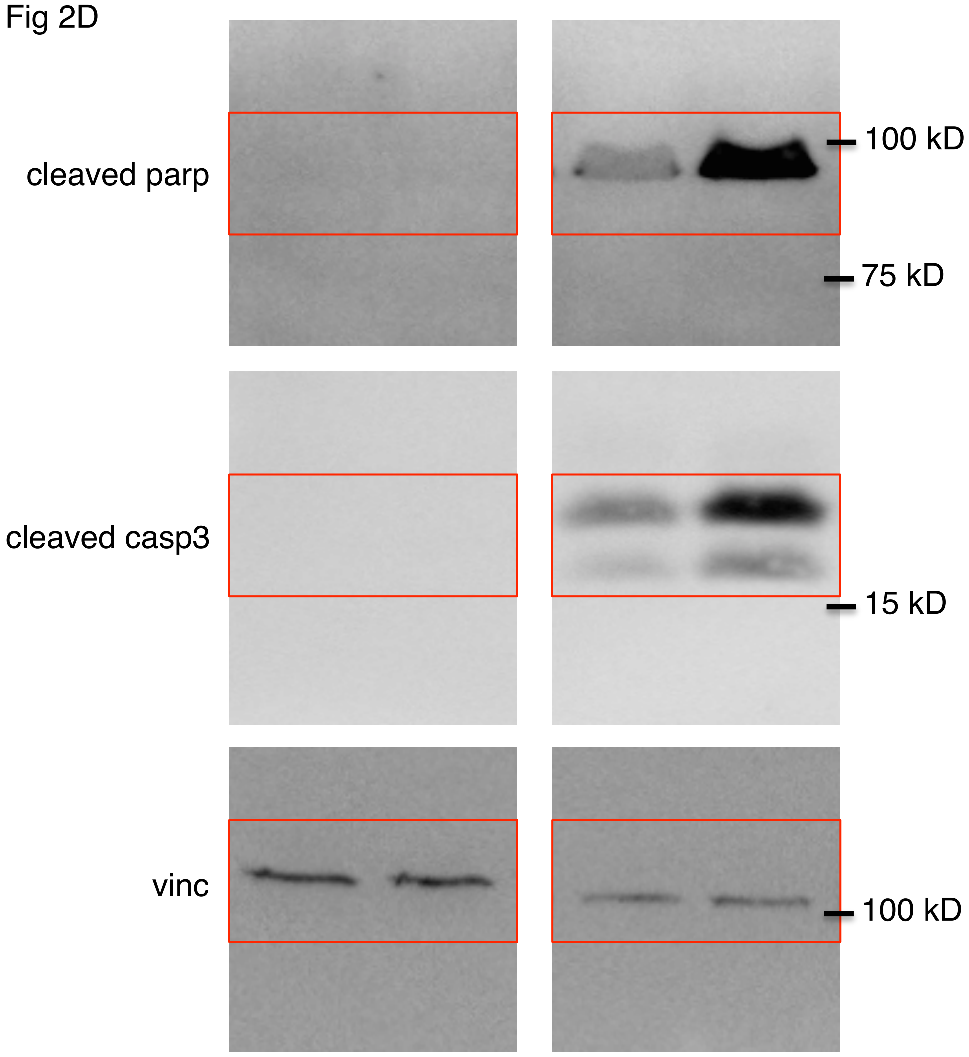
cleaved parp
100 kD
75 kD
cleaved casp3
15 kD
vinc
100 kD

Fig 2E

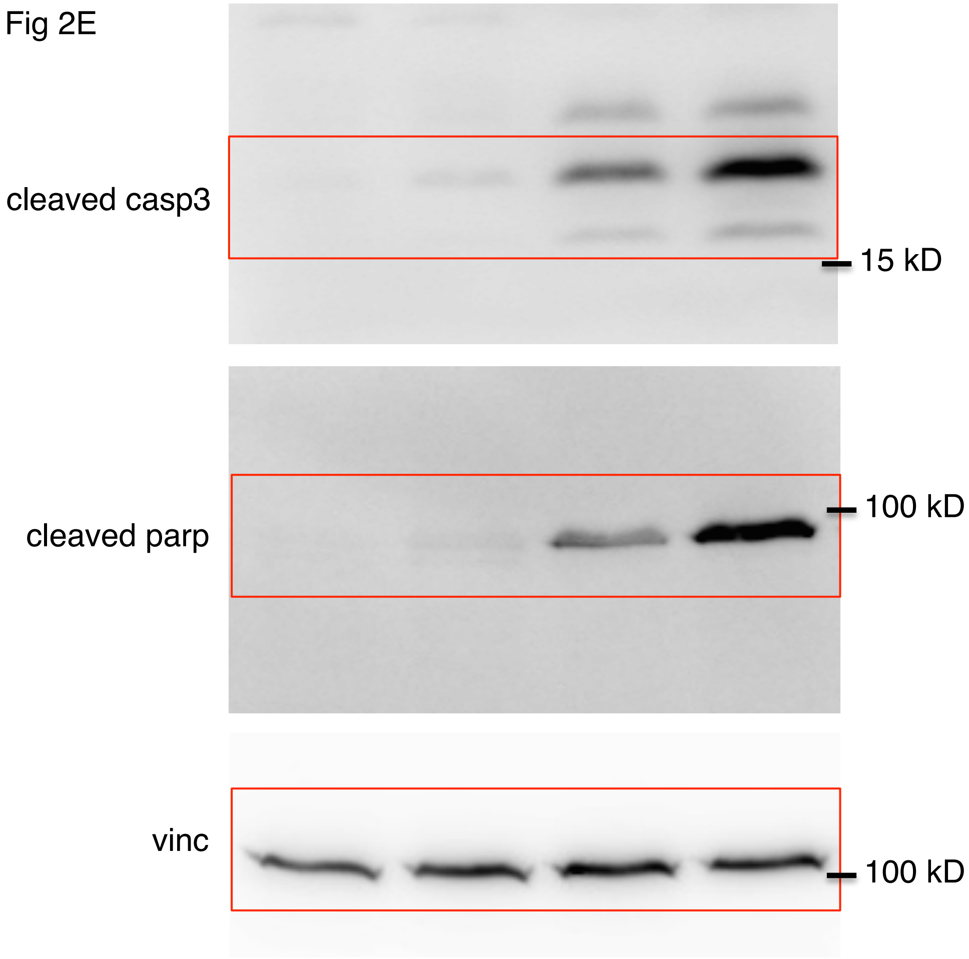
cleaved casp3
15 kD
cleaved parp
100 kD
vinc
100 kD

Fig 2F

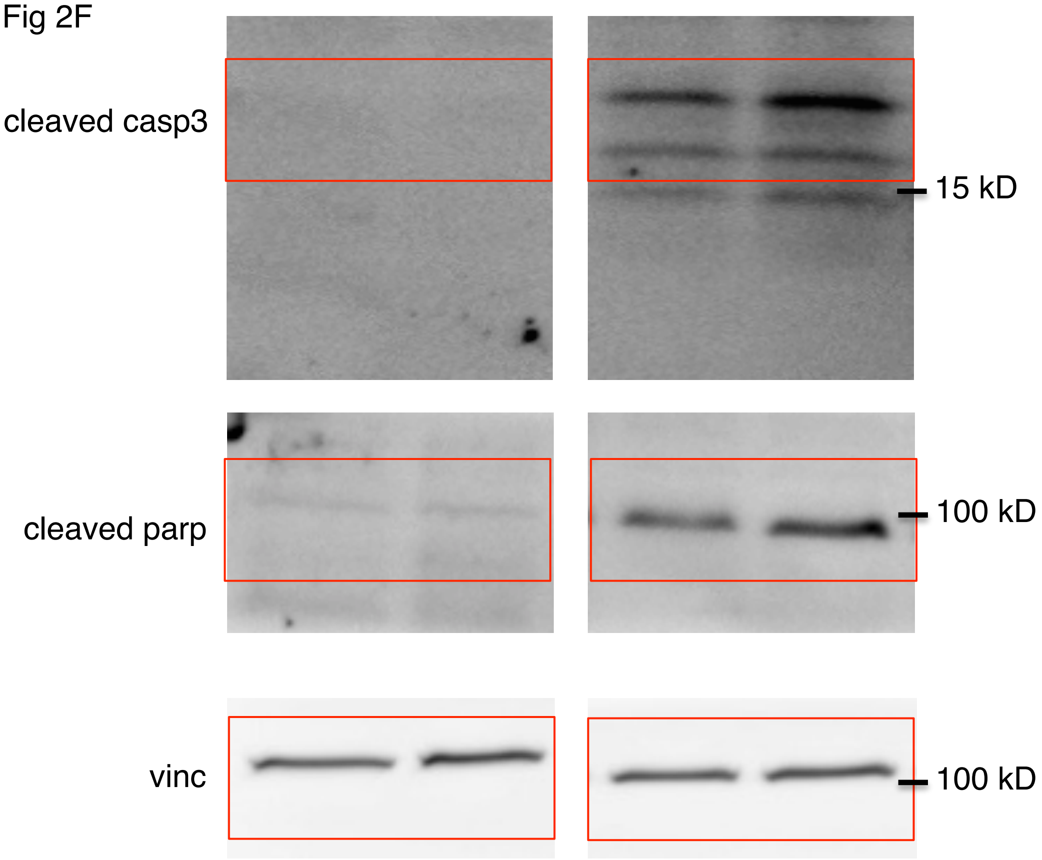
cleaved casp3
15 kD
cleaved parp
100 kD
vinc
100 kD

Fig 2G

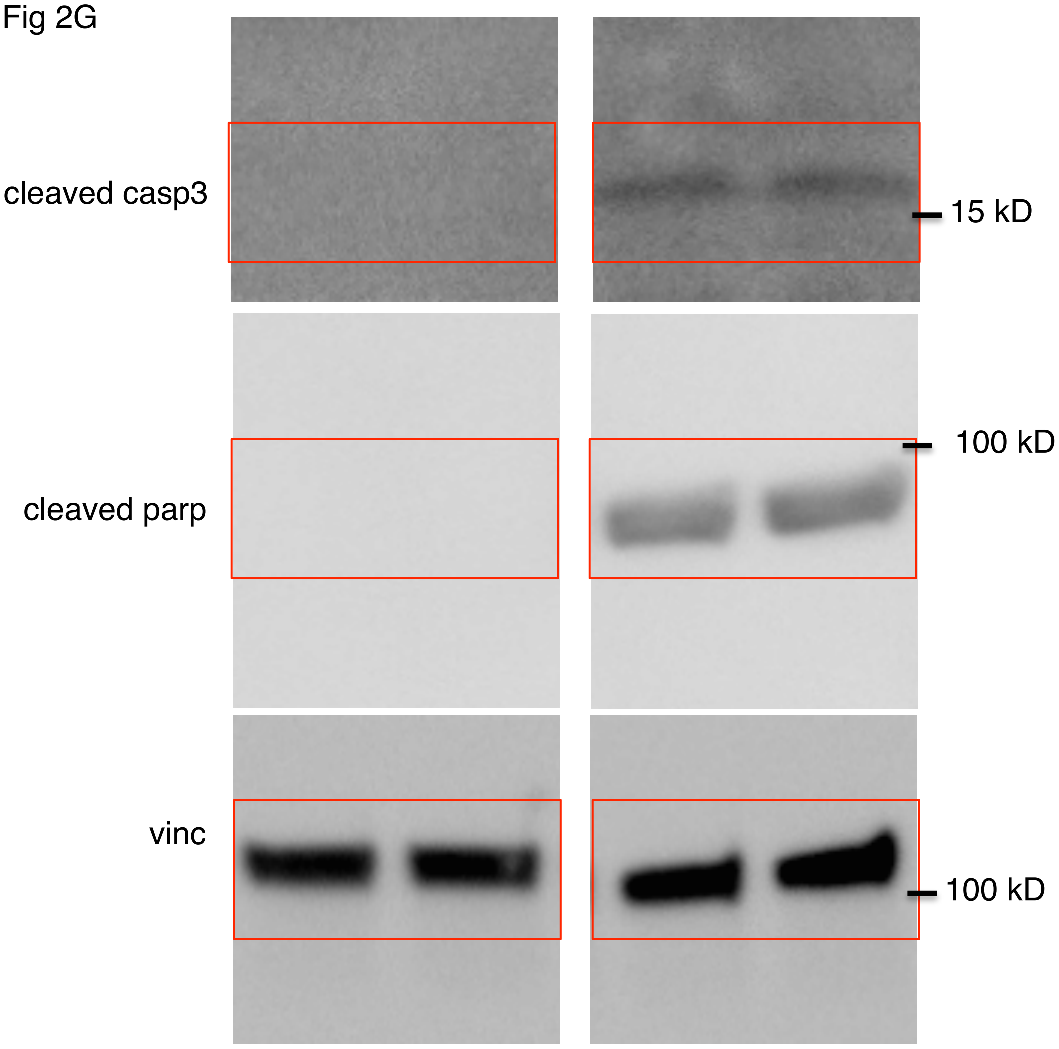
cleaved casp3
15 kD
100 kD
cleaved parp
vinc
100 kD
